# Global phosphorus shortage will be aggravated by soil erosion

**DOI:** 10.1038/s41467-020-18326-7

**Published:** 2020-09-11

**Authors:** Christine Alewell, Bruno Ringeval, Cristiano Ballabio, David A. Robinson, Panos Panagos, Pasquale Borrelli

**Affiliations:** 1 Environmental Geosciences, Department of Environmental Science, University of Basel, Basel, Switzerland; 2 ISPA, Bordeaux Sciences Agro, INRAE, 33140 Villenave d’Ornon, France; 3 European Commission, Joint Research Centre, Ispra, Italy; 4 UK Centre for Ecology & Hydrology, Environment Centre Wales, Bangor, United Kingdom; 5 Department of Biological Environment, Kangwon National University, Chuncheon-si, Gangwon-do Republic of Korea

**Keywords:** Biogeochemistry, Geomorphology, Sedimentology, Agriculture, Geography

## Abstract

Soil phosphorus (P) loss from agricultural systems will limit food and feed production in the future. Here, we combine spatially distributed global soil erosion estimates (only considering sheet and rill erosion by water) with spatially distributed global P content for cropland soils to assess global soil P loss. The world’s soils are currently being depleted in P in spite of high chemical fertilizer input. Africa (not being able to afford the high costs of chemical fertilizer) as well as South America (due to non-efficient organic P management) and Eastern Europe (for a combination of the two previous reasons) have the highest P depletion rates. In a future world, with an assumed absolute shortage of mineral P fertilizer, agricultural soils worldwide will be depleted by between 4–19 kg ha^−1^ yr^−1^, with average losses of P due to erosion by water contributing over 50% of total P losses.

## Abstract (web summary)

Phosphorus is an essential nutrient critical for agriculture, but because it is non-renewable its future availability is threatened. Here the authors show that across the globe most nations have net losses of phosphorus, with soil erosion as the major route of loss in Europe, Africa and South America.

## Introduction

Phosphorus (P) being a key element in DNA, RNA as well as ATP and phospholipids is essential for the growth, functioning and reproduction of all life on earth. In natural ecosystems the P that is lost from the soil-plant cycling system has to be replaced by the slow process of rock weathering^1^ or added via fertilizer in human managed systems (there is no equivalent to the biological N_2_ fixation which is only kinetically limited but potentially not resource limited). However, if fertilization with animal waste or human excreta is not available or not organized, P fertilizers stem from non-renewable geological P deposits, which are an increasingly limited resource (this is again in contrast to N, as N fertilizer can be produced as an endless resource via the Haber Bosch process as along as energy and natural gas is available). The one-way flow of P from mineral reserves to farms (e.g., soils), to freshwaters and finally into oceans, are already considered to be beyond the safe operating space for sustainable human development^2^. The potential threats of global P limitation due to peak phosphorus have been discussed intensively in the recent past^3–7^. The imminent threat of such a P limitation has been restrained somewhat as obviously some P deposits had been overlooked or misclassified in the past which will theoretically last for the next 600 years of global P supply^8^. However, the socio economic as well as political consequences are still dramatic with the newly discovered P reserves being restricted to a small region of the Western Sahara and Morocco. Recent literature is controversial as to whether or not P supply from rock reserves in the next decades will be a real physical scarcity^5^ or will be limited by economic and technical constraints^6,7,9^. Ulrich and Frossard^10^ argue that the main problem is not the geological P availability, but rather socio-economic (e.g., fertilizer access) or environmental (e.g., water pollution) vulnerabilities, resulting from current and future P production and consumption patterns. In parallel to the 2007-2008 global food crisis, phosphate rock and fertilizer demand exceeded supply, and prices increased by 400% within a 14-month period^11^ demonstrating the sensitivity of this market. The following consequences have been instances of farmers riots and death due to severe national shortage of fertilizers in countries such as India, which are totally dependent on phosphate imports^3,12^. The growing demand for P fertilizer globally has caused an increase in the cost of rock phosphate from about $80 per U.S. ton in 1961 to $700 per ton in 2015 (with large year-to-year fluctuations)^13,14^.

The most crucial potential P loss (P_Loss_) from ecosystems is loss from the soil due to soil erosion by water^2^, and this often happens in sudden events^15^. As the major part of soil P is tightly sorbed to mineral particles, bound within organic matter or precipitated as poorly soluble salts, it is mainly exported from soils to water bodies via erosion by water. Only a very small fraction of P in soils and bedrocks is available to plants or might leach as dissolved soluble phosphate^16–18^ with the exception of excessively fertilized soils with increasing P availability^19,20^.

**Fig. 1:**
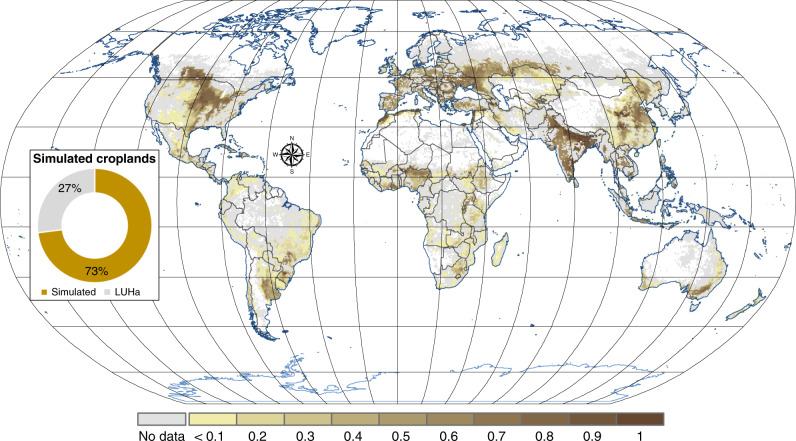
Modeled area and global distribution of cropland. The chromatic scale represents the cropland ratio fraction of cropland from total land per pixel of the modeled land. The gray color indicates the cropland areas that were excluded from the modeling due to data unavailability. The insert panel illustrates the simulated versus total cropland area according to the harmonization of land-use LUHa.v1 database^73^.

Recently, global modeling approaches demonstrated that agricultural management aiming at closing nutrient cycles such as increasing P use efficiency and/or recycling of animal manure and human excreta, might have a major impact on P balances^1,21^. In considering principal agronomic P inputs and outputs but neglecting soil erosion MacDonald et al.^22^ calculated that 29% of the global cropland area had P deficits and 71% had surpluses (data of the year 2000). Opportunities for recovering phosphorus and reducing demand have been discussed extensively^3,12,23^. However, most global, continental or regional evaluations of the P cycle have not calculated P loss due to soil erosion, even though erosion is acknowledged as clearly contributing to potential P loss^3,21,22,24–26^, or soil erosion is very simplistically addressed without spatially discrete data analysis^2,16,27–30^ (e.g., by either extrapolating erosion rates of Pimentel^31^ or Smil^16^ to a global scale or using FAO average statistics (FAOSTAT http://faostat3.fao.org)). Nevertheless, there is general agreement, that P losses due to soil erosion are a crucial parameter in the global P cycle, but that at the same time estimation of those rates are connected to a high uncertainty^15,16,27,28^, owing to the uncertainty in spatially distributed soil erosion rates and/or the limited availability of global soil phosphorus data^15^. The neglect, or the only rough estimates of soil erosion in evaluating P cycling in the past were simply due to a lack of available spatially discrete data. Here, we overcome these limitations by specifying the P loss due to soil erosion by water in matching the global spatially discrete soil erosion maps of Borrelli et al.^32^ with spatially distributed global P content of soils for croplands from Ringeval et al.^33^ to develop and assess global soil P loss maps and balances from arable land (Fig. 1). Our main finding is that the loss of P in agricultural systems, especially in areas with low or no future P fertilizer input, depends on soil erosion and poses an imminent threat to system functioning.

## Results and discussion

### Global P losses from soils and soil P balances

**Fig. 2:**
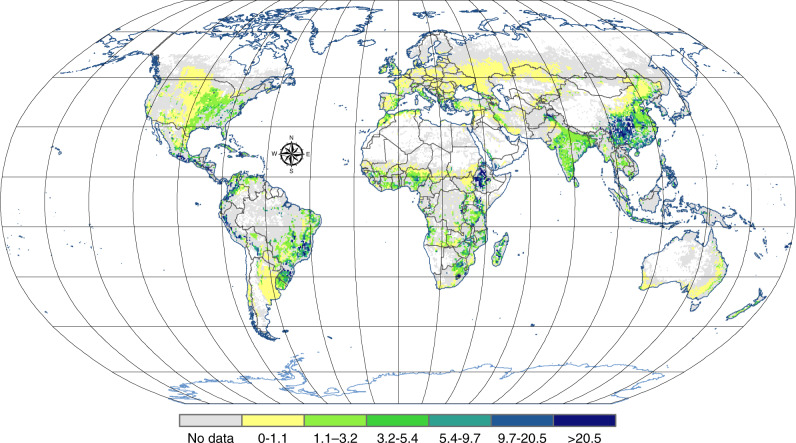
Global average phosphorus (P) losses due to soil erosion in kg ha^−1^ yr^−1^. The chromatic scale represents the P losses estimates, while the gray color indicates the cropland areas that were excluded from the modeling due to data unavailability. Note that classes are not regularly scale ranked but are divided into six classes using the quantile classification method. Only plant available fractions were considered. For the more residual P fractions please refer to Table 1 or Figs. 3 and 4).

**Fig. 3:**
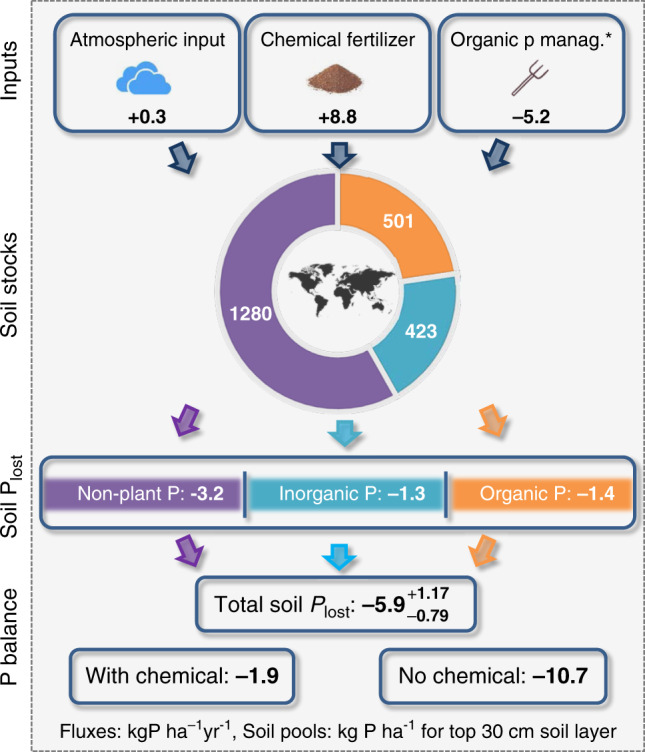
Global P soil pools and depletion due to erosion. Arrows indicate fluxes (positive: net input to soils, negative: depletion of soils). *Organic P management = sum of manure and residue input minus plant uptake. Non-plant P = non-plant available P. Inorganic and organic P give plant available fractions. Total soil P: sum of P fractions lost from soil via erosion with relative errors. No/with chemical = P balance with and without chemical fertilizer.

**Fig. 4:**
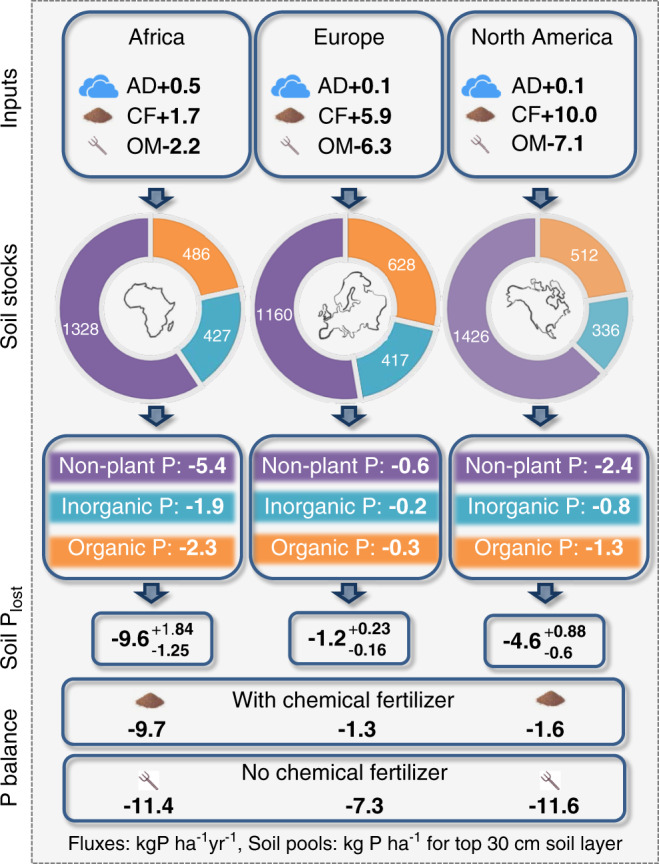
Soil P pools and depletion due to erosion in Africa, Europe and North America. AD = Atmospheric Deposition. CF = Chemical Fertilizer. OM = Organic P management = sum of manure and residue input minus plant uptake. Arrows indicate fluxes (positive: net input to soils, negative: depletion of soils). Non-plant P = non-plant available P. Inorganic and organic P give plant available fractions. Soil P_lost_: sum of P fractions lost from soil via erosion with relative errors. No/with chemical = P balance with and without chemical fertilizer.

All continents result in negative P balances (e.g., net P losses from agricultural systems, Table 1; Fig. 2) except Asia, Oceania and Australia, with Asia having a slightly positive but near zero P balance. This is in spite of high to very high chemical fertilizer inputs (with a range of 1.7 to 13 kg ha^−1^ yr^−1^ between the different continents, with national values reaching up to 14 and 19 kg ha^−1^ yr^−1^ for the European Union 15 old Member States (EU15, member states joining before 2004 mainly in western and northern Europe) and China, respectively). Most negative P balances are indicated for Africa due to very low chemical fertilizer input of 1.7 kg ha^−1^ yr^−1^ paired with high losses due to soil erosion of 9.6 kg ha^−1^ yr^−1^. South America as well as Central and Eastern Europe (NEU11; which are the new member states joining the EU after 2004 with the exception of Cyprus and Malta) also exhibit high P losses but for different reasons. South America has a very high chemical fertilizer input but also high losses due to soil erosion paired with high P exports due to organic P management (calculated as the sum of manure and residue input minus plant uptake). In contrast, the eastern European Union New Member States (NEU11) have rather low erosional losses but also very low chemical fertilizer input. With the hypothetical assumption of no replenishment due to chemical fertilizer (e.g., due to economic or technical constraints), calculation of soil P balances results in negative balances globally, as well as for all the continents and regions considered (depletion between 4 and 20 kg P ha^−1^ yr^−1^; Figs. 3 and 4; Table 1). The latter demonstrates the vulnerability of today’s global land management system and its strong dependency on chemical P fertilizers from non-renewable mineable P deposits.

**Table 1:** Main phosphorus (P) statistics in kg P ha^−1^yr^−1^ for all continents and selected countries.

	Global	Africa	North America	South America	Geographic Europe	EU15^a^	NEU11^b^	Asia	China	Oceania	Australia
Atmospheric input	0.3	0.5	0.1	0.1	0.1	0.1	0.2	0.5	0.5	0.1	0.2
Chemical fertilizer	8.8	1.7	10.0	11.4	5.9	13.7	5.2	11.9	19.0	12.7	10.4
Organic P management	−5.2	−2.2	−7.1	−8.7	−6.3	−12.2	−8.5	−3.9	−7.6	−2.5	−4.1
Erosion from:											
Non-plant avail. P^c^	−3.2	−5.4	−2.4	−5.1	−0.6	−0.9	−0.7	−4.1	−6.4	−0.9	−0.5
Plant avail. inorganic P^c^	−1.3	−1.9	−0.8	−1.8	−0.2	−0.5	−0.2	−1.8	−2.8	−0.6	−0.2
Plant avail. organic P^c^	−1.4	−2.3	−1.3	−2.0	−0.3	−0.7	−0.3	−1.8	−3.2	−0.3	−0.2
Total soil P	−5.9	−9.6	−4.6	−8.9	−1.2	−2.1	−1.2	−7.8	−12.3	−1.8	−0.9
Balance^d^	−1.9	−9.7	−1.6	−6.1	−1.3	−0.4	−4.3	0.6	−0.4	8.5	5.6
Balance – Chem. Fert^e^	−10.7	−11.4	−11.6	−17.4	−7.3	−14.1	−9.5	−11.3	−19.5	−4.2	−4.8
Erosion loss (%)	$54.6_{7.0}^{10.4}$	$84.5_{10.6}^{15.6}$	$39.5_{5.1}^{7.5}$	$50.8_{6.5}^{9.6}$	$15.8_{2.0}^{3.0}$	$15.0_{1.9}^{2.8}$	$12.7_{1.6}^{2.4}$	$69.3_{8.7}^{12.8}$	$63.3_{8.0}^{11.9}$	$43.7_{5.5}^{8.1}$	$19.1_{2.4}^{3.6}$

Organic P management = sum of manure and residue input minus plant uptake. Total soil P = total P depletion of soils due to soil erosion by water globally. Erosion loss (%) = Ratio of erosion losses to soil P balance excluding chemical fertilizer (balance—chemical fertilizer) with non-symmetric to the mean uncertainty estimates in %. Positive numbers give net retention in soils, negative depletion of soils. Geographic Europe includes European parts of the former Soviet Union.

^a^EU15 (Austria, Belgium, Denmark, Finland, France, Germany, Greece, Ireland, Italy, Luxembourg, Netherlands, Portugal, Spain, Sweden, United Kingdom)

^b^NEU11: Central and eastern European countries joining the EU after 2004: Bulgaria, Croatia, Czech Republic, Estonia, Hungary, Latvia, Lithuania, Poland, Romania, Slovak Republic, and Slovenia

^c^see the definition of plant available and non-plant available soil pools in the methods section following Hedley et al.^38^ and Helfenstein et al.^41^

^d^Balance = atmospheric input + chemical fertilizer + organic P management + total soil P losses due to soil erosion

^e^Balance—Chem. Fert. = hypothetical Balance without chemical P fertilizer input

Our area related calculations result in an average P loss for arable soils, due to erosion by water, of approximately $5.9_{ - 0.79\% }^{ + 1.17\% }$ kg ha^−1^ yr^−1^ globally (Fig. 3, Table 1). This is around 60% of the rates given by Smil^16^ who estimated 10 kg P ha^−1^ yr^−1^ from arable fields due to soil erosion by water. Our total P losses due to soil erosion by water from arable soils globally result in 6.3 Tg yr^−1^ with 1.5 Tg yr^−1^ for organic and 4.8 Tg yr^−1^ for inorganic P. With these values the results are at the lower end of the range discussed in the literature (between 1–19 Tg yr^−1^, Table 2).

**Table 2:** Global fluxes from soils/arable systems to waters as discussed in recent literature. For comparability, all values were normalized to 1 billion ha of arable land (with original values given in brackets).

	Area considered	Tg yr^−1^ normalized to 1 billion ha	Method to calculate soil erosion
Meybeck et al.^78^	All terrestrial land	14 (20)	Based on global particulate P river export to coastal seas calculated from global ratios of particulate carbon to phosphorus
Smil^16^	World’s croplands in the mid-1990s	9–10.5 (13–15)	Global erosion rates from Smil^79^, no further details
Mackenzie et al.^80^	All terrestrial land	15.4 (22)	Terrestrial Ocean Atmosphere Ecosystem Model
Liu^34^	The world’s croplands (1.5 billion ha)	12.8 (19.3)	Extrapolating erosion rates from Pimentel^31^ to global levels
Bouwman et al.^1^	Cropland area (1.54 billion ha)	0.7–1.3 (1–2)	Global NEWS Model based on FAO statistics
Cordell et al.^3^	Arable soils	5.6 (8)	No information on methods or exact area given but P flux due to erosion illustrated in figure
Quinton et al.^15^	Agricultural land	10.2–18.5 (14.6–26.4) Organic: (2.1–3.9) Inorganic: (12.5–22.5)	Based on erosion rates by Van Oost et al.^71^ considering water and tillage erosion; no information given, how organic versus inorganic was specified
Chen and Graedel^28^	Agricultural land 1.1 billion ha	8.2–12 (9–13.2)	Erosion rates based on Liu^34^
This study	1.04 billion ha of arable land	6.3 (6.3) Organic: 1.5 Inorganic: 4.6	Erosion rates based on Borrelli et al.^32^, organic (sum of labile and stable organic) and inorganic P (as sum of labile and inorganic P bound to secondary minerals plus occluded and apatite P) species according to Hedley fractionation^33^

Liu^34^ in estimating net input to, and output from, cropland systems indicated a net loss of P from the world’s croplands of about 12.8 Tg  yr^−1^ (calculating input from atmosphere, weathering and chemical fertilizer versus output from organic P management, soil erosion and runoff), which would be, according to their calculations, the same order of magnitude as synthetic fertilizer input (13.8 Tg yr^−1^ for statistical year 2003/2004). Our calculations result in an approximate net soil loss due to erosion of 6.3 Tg yr^−1^ with a global average chemical fertilizer input of 9.2 Tg yr^−1^. The fluxes clearly show the critical dependence on chemical fertilizer globally, with a hypothetical net-average-area related depletion of 10.7 kg ha^−1^ yr^−1^ globally without the compensation due to chemical fertilizer, from which a loss of 5.2 kg ha^−1^ yr^−1^ stems from organic P management (sum of manure and residue input minus plant uptake) and 5.9 kg ha^−1^ yr^−1^ from soil erosion (Table 1). Continental and national erosional P losses are between 40 and 85% of total P losses from agricultural systems with the exception of Europe and Australia (16 and 19%, respectively). Globally, as well as for Africa, South America and Asia, P soil losses due to erosion are higher than losses due to organic P management.

A recent quantification of atmospheric P dust input, based on dust measurements in the Sierra Nevada, concluded that measured dust fluxes are greater than, or equal to, modern erosional outputs and a large fractional contribution relative to bedrock^35^. However, even though their measured and modeled maximum atmospheric P fluxes were in the same order of magnitude as the atmospheric flux data of Wang et al.^36^ used in this study (0.1 kg ha^−1^ yr^−1^ for North America), erosion rates from sediment trap measurements in their investigated forest ecosystems were considerably lower (maximum of 0.06 kg ha^−1^ yr^−1^^35^) than might be expected in arable lands worldwide. As such, we can clearly not agree with the above conclusion.

Mitigation of the soils P status in the long term by decreasing the deficits of the current organic P management seems difficult and rather unlikely in many regions of the world (see discussion of continental balances below). As such, and considering the expected shortage of P supply from industrial fertilizers in the future, the evaluation of P fluxes clearly shows that soil erosion has to be limited to the feasible absolute minimum in the future.

Only a small fraction of the total soil P is plant available, because a large fraction is either bound in, adsorbed to, or made unavailable by occlusion in minerals (apatite and occluded P)^37^. Our modeling approach follows the approach of Yang et al.^37^ that builds on existing knowledge of soil P processes and data bases to provide spatially explicit estimates of different forms of naturally occurring soil P on the global scale. Yang et al.^37^ uses data acquired with the Hedley fractionation method^38^ which splits soil P into different fractions that are extracted sequentially with successively stronger reagents and which are merged into various inorganic and organic pools. There is great uncertainty to associate these fractions with functional plant uptake^39,40^ but some recent work aims at quantifying residence times of the different fractions^17,41^. We present the total P loss as well as the plant-available pool (Figs. 3 and 4, Table 1) as the most labile P which can participate to plant nutrition in a time scale up to months^17^ (i.e., the so-called labile inorganic P, inorganic P bound to secondary minerals, labile and stable organic P^33,37^). We contrast these more labile, short lived fractions with fractions considered very stable which would not be plant available at short time scales (i.e., inorganic P associated with minerals such as apatite or occluded P^17^, corresponding to 52% of total soil P in our estimates at the global scale).

### Verification of soil P loss with a comparison to riverine P exports

Verification of our proposed P soil losses might be done indirectly via a comparison to riverine P loads. However, to verify our on-site P soil loss approach with off-site P river export data requires two prerequisites: (i) an estimation of the agricultural erosion and runoff contribution to total P loads in the rivers (as the latter will be the total sum of agricultural, urban and industrial runoff) and (ii) an assumption on sediment delivery rates (e.g., the percent of sediments reaching the rivers from the total eroded sediments) to estimate P loads to rivers from on-site P soil loss. Regarding the first prerequisite, we searched for published river P export data with a separation of the agricultural erosion and runoff contribution from total P loads in the rivers. Regarding the second prerequisite, there is a lack of models describing integrated sediment-delivery to rivers on a continental or global scale, but it has been discussed that sediment delivery ratios generally decrease as drainage area increases, ranging from roughly 30–100% in small catchments (≤0.1 km^2^) to 2–20% at large spatial scales (e.g., ≥1000 km^2^)^42^. For our comparison, we used a range of average sediment delivery rates between 11–30% as used in recent large scale studies from continental to global scale^43,44^. In doing so, we would like to point out, that even if 70–89% of the P lost from agricultural soils might be re-deposited within catchments, potential threats of P loss from soils are not reduced. Erosion (and thus P loss) occurs predominantly on agricultural soils while re-deposition will mostly occur in depositional hollows, wetlands, riparian zones or buffer strips. Thus, P is lost as a nutrient on food and feed production sites but re-deposited as a potential ecological threat to biodiversity and ecosystem health due to its eutrophication effect in less intensively or unmanaged ecosystems. Last but not least we would like to point out that RUSLE only considers soil displacement due to rill and inter-rill erosion neither considering tillage and gully erosion nor land sliding.

A comparison of calculated potential P export to rivers from our on-site soil P losses is well within the range of published riverine P exports (Table 3). Beusen et al.^45^ used total suspended sediment measurements from the GEMS-GLORI database to extrapolate spatially distributed sediment rates for the world’s largest rivers with land use, topography, lithology and precipitation as factors in a multiple linear regression approach accounting for soil erosion as well as sediment trapping. The associated nutrient exports for all continents, as well as global assessment were made by calibrating nutrient export to sediment rates with the model Global News^45^ using established correlations between sediment and nutrient concentrations. In comparing their P export with suspended sediments in rivers^45^ to our assessments, we underestimated P export globally as well as for all continents, with the exception of Africa (Table 3), which is, however, (i) strongly related to assumed sediment delivery rates and (ii) our RUSLE application only considering rill and inter-rill erosional processes with an unknown contribution of gullies, landslides and tillage erosion.

**Table 3:** On-site P loss from gross soil erosion (this study), calculated potential riverine loads with sediment delivery ratios between 11–30%^43,44^ and comparison to global and regional riverine P export studies. All values in kg P ha^−1^yr^−1^.

Continent	P soil loss (this study)	River loads (this study)		River loads global study	River loads regional values	Origin of data for regional studies	Reference of regional studies
		11%	30%	Beussen et al.^45^			
Africa	9.6	1.1	2.9	1.9	1.8 - 2.2	Average for sub-Saharan Africa, years 1982 and 2000; estimated from nutrient soil measurements and measured soil erosion rates	Stoorvogel et al.^50^
North America	4.6	0.5	1.4	1.5	0.3–1	143 catchments across the US; modeled P partitioning with measured P fluxes in rivers	Stackpoole et al.^47^
					1.6	Total P loads to Lake Erie including industrial and urban runoff, 38507 km^2^ as average of the loads from the three catchments Maumee, Sandusky, Cuyahoga	Baker et al.^48^
South America	8.9	1.0	2.7	3.9	2.4	Measurement at outlet of Guayas Basin, Ecuador, 32026 km^2^	Borbor-Cordova et al.^51^
Geographic Europe	1.2	0.1	0.4	0.6	0.05–1.5	Estimated P export from agricultural runoff for 17 large scale European catchments	Kronvang et al.^46^
Asia	7.8	0.9	2.3	4.0	—		
China	12.3	1.4	3.7	—	2.7	Yangtze River Basin (1.8 Million km^2^ equivalent to 18.8% of the Chinese Territory), modeled P partitioning with measured P fluxes in rivers to assess P load from agricultural fields	Wang et al.^49^
Oceania	1.8	0.2	0.5	2.6	—	—	
Australia	0.9	0.1	0.3	—	0.3–0.5	Stream monitoring in three catchments between 241 and 308 km^2^	Koci et al.^81^
Global	5.9	0.7	1.8	2.7	—	—	

An analysis of 17 large scale European catchments (250–11,000 km^2^) quantifying the loss of P to surface waters from an off-site perspective (P flux measurement in waters) resulted in 0.05–1.5 kg ha^−1^ yr^−1^ of P loss due to soil erosion and runoff from agricultural lands into streams and rivers (excluding a Greek catchment with a very high P export of 6 kg ha^−1^ yr^−1^)^46^. Recalculating our on-site soil P losses with sediment delivery ratios between 11–30% results in rates for geographic Europe between 0.1–0.4 kg ha^−1^ yr^−1^ which are at the lower end of the range of Kronvang, et al.^46^ (Table 3). Potential P losses as riverine export due to agricultural runoff and erosion of 143 watersheds across the U.S.^47^ are in the same range as the calculated P loss assessment of our study, while P loads to Lake Erie assessed in a regional study^48^ seem slightly higher (Table 3). However, no partitioning between agricultural, urban or industrial fluxes was possible from the latter study. An assessment of the Yangtze River Basin (with 1.8 Million km^2^ near 20% of the whole Chinese territory) gives modeled soil P losses (on-site perspective) between 0–196 kg ha^−1^ yr^−1^ demonstrating the huge spatial heterogeneity of on-site soil erosion rates^49^. The model output was calibrated with total measured nutrient loads in rivers, while the partitioning differed between dissolved point and non-point as well as adsorbed non-point pollution which can be mostly attributed to soil erosion of agricultural fields. The average P loads due to soil erosion from agricultural fields in the Yangtze River Basin compares well with the range assessed in our study for China (2.7 versus a range of 1.4 to 3.7 kg ha^−1^ yr^−1^, respectively). The same holds true for assessment comparisons of Africa^50^ and South America^51^ (Table 3), even though it should be considered that especially for these latter studies scale differs considerably from our approach.

### Regional P losses and balances

Parallel to the distribution pattern and dynamics of global soil erosion by water^32^, P losses from soils due to water erosion are most dramatic in countries and regions with intensive agriculture and/or extreme climates (e.g., droughts followed by significant rain events or high frequencies of heavy rain storms) due to high erosivity effects^52^. As such, our calculations result in extremely high P losses due to erosion (>20 kg ha^−1^ yr^−1^) in regions such as eastern China, many regions in Indonesia, parts of east and south-eastern Africa (Ethiopia, Eritrea, Mozambique), Central America and parts of South America (South-Eastern Brazil; Southern Chile, Peru (Fig. 2)). A very high P loss (10 to 20 kg ha^−1^ yr^−1^) is estimated for parts of Southern Africa (South Africa, Madagascar, Tanzania) and South America (Bolivia) and a high loss (5–10 kg ha^−1^ yr^−1^) for most of India, as well as regions in Southern Africa (Angola, Zambia) and South America (Uruguay) (Fig. 2). Even though the underlying erosion model algorithm does not calculate the net catchment output but rather the on-site displacement of soil sediments which might then be re-located to other parts of the fields or even buried at depositional places, the considered on-site field management will clearly be confronted with substantial P losses due to soil erosion by water. Only considering agronomic P inputs and outputs without including P losses due to erosion by MacDonald et al.^22^ resulted in a very different global P pattern: most widespread large deficits were in South America (North-Eastern countries, e.g., Argentina and Paraguay), the northern United States and Eastern Europe while the largest surpluses covered most of East Asia, Western and Southern Europe, the coastal United States, South-Eastern Brazil and Uruguay.

With average soil depletion due to erosion of 9.6 kg ha^−1^ yr^−1^ in Africa, the overall P balance is already negative by 9.7 kg ha^−1^ yr^−1^ today (Table 1, Fig. 4). As the average P depletion in Africa due to negative fluxes in organic P management equals the input fluxes from the atmosphere plus chemical fertilizer, African farmers could decrease P losses to near zero with effective soil erosion mitigation. Even though the system’s P depletion due to organic P management is relatively low in Africa (−2.2 kg ha^−1^ yr^−1^) compared to a global average (−5.2 kg ha^−1^ yr^−1^), the high overall P losses are unlikely to be covered neither from a mitigated and more sustainable organic P management nor from increased chemical fertilizer input. P fluxes due to organic P management are calculated here as the sum of manure and residue input minus plant uptake (which results in biomass export in arable systems with the exception of residues left on the field). The overall sum of plant uptake is likely to increase with increased need for food and feed parallel to a predicted population and livestock growth in Africa in the future. Many soils in sub-Saharan Africa have already been characterized as deficient for levels of plant-available P for the last decades^53^. Manure and residue input is simultaneously in demand in Africa today (shortage of biomass in general, low animal production and even if there is manure available, there are no means to transport it to where it is needed), which results in the recommendations of an integrated farm management with combinations of organic and inorganic fertilizers^54–56^. With the inorganic P fertilizers becoming increasingly scarce, the depletion due to organic P management can be expected to increase in Africa in the future. Simultaneously, today’s prices for chemical fertilizer can already be 2–6 times more expensive for a farmer in Africa than in Europe due to higher transport and storage costs^3^, even though Africa itself has the highest geological P deposits in the world (according to today’s estimates 80% of the global geological P deposits are located in Morocco and the Western Sarah^8^). As such, and if the political situation does not change dramatically (e.g., that the P supplies are marketed within Africa instead of being exported to US, Europe and China), the only realistic means of reducing P depletion of African soils today, and in the future, is to drastically reduce soil erosion.

We recognize that the values calculated in Table 1 and Fig. 4 are gross estimates over large scales and that spatial context and scale, especially on the African continent is important. P deficiency is a country, district, farm and soil specific issue in Africa, for example parts of east Africa and the Sahel have substantial deficiencies^57^. In sub Saharan Africa ~40% of soils are considered to have low nutrient reserves (<10% weatherable minerals) and soil degradation is enhancing the deficiencies^58^. Erosion control is important, but is only part of the solution, which needs to be multifaceted. Omuto and Vargas^59^ assessed total soil erosion based on field measurements in Malawi to have increased ~10% between 2010 (26 t ha^−1^ yr^−1^) and 2017 (30 t ha^−1^ yr^−1^, note that erosion rates from rill and interill erosion only in our modeled assessments for Malawi based on Borrelli et al.^32^ are 19 t ha^−1^ yr^−1^). Surveys of farmers indicated that 45% were not investing in soil erosion control and many of these were in areas where there was a high level of need. Moreover, farmers recognized that the lack of implementation of sustainable land management practices was a main reason for high erosion rates, over and above the fact that the soils are often vulnerable and fragile. Hence, erosion control offers part of the solution.

Attempts at increasing nutrient status in Malawi since 2010 have, through blanket mineral fertilizer recommendations, not only yielded significantly higher production transforming the country into a food-exporting nation, but also led to soil acidification in many districts^59^. As such, in addition to soil erosion control integrated soil fertility management (ISFM) is required that combines using mineral fertiliser and organics as well as growing legumes (leguminous trees and/or cover crops)^57^. Even though leguminous trees offer the potential to access nutrients deep in the subsoil and deposit them to the surface via litter fall, the time delay required to implement such a system acted as a barrier to adoption, with farmers having to forgo one crop^57^. In addition, the low P status of soils in many African regions^57^ means that recycling of organic materials is insufficient to boost yields, hence the need for mineral fertiliser. Even though leguminous plants will primarily improve N and not P status of soils, a recent review on leguminous crops found that grain legumes can access less accessible forms of P under P-deficient conditions (through the release of root exudates; access to more of the labile P through a finer root architecture, and enhanced associations with mycorrhiza)^60^. Hence integrated, region and soil specific management options are required and these complexities have to be held in mind when interpreting our continental scale figures, which might help provide global context for action. While the complexities of P management are well documented and discussed^57–60^ we would like to draw the attention to mitigating soil erosion as one important part towards decreasing malnutrition in Africa.

With high soil depletion rates due to erosion of 8.9 kg ha^−1^ yr^−1^ in South America and losses due to organic P management of 8.7 kg ha^−1^ yr^−1^ the P balance of land under arable use results in a negative balance of −6.1 kg ha^−1^ yr^−1^ in spite of the current high chemical fertilizer input of 11.4 kg ha^−1^ yr^−1^ (Table 1). As South America has no notable geological P deposits, continuing high or even increasing fertilizer application to balance high soil P losses seems unrealistic in the future (and would also be unacceptable from an ecological perspective as long as soil erosion rates and thus P output to fresh and ocean waters is not substantially reduced). However, with a much higher continental biomass production capacity compared to Africa, many regions in South America might be able to decrease P losses due to improved organic P management (e.g., by generally applying conservation agriculture, organic and/or other sustainable farming practices^61^) and/or increasing use of animal waste or human excreta^1^, or applying management systems with increased use of residue input. Nevertheless, in the long-term a reduction of P losses due to mitigating soil erosion (e.g., conservation agriculture, mulching, increased vegetation cover, intercropping, topography adapted land management) will be the most efficient way and will simultaneously increase soil health, the general nutrient status of soils and water retention capacity as well as decreasing the ecologically negative impact on fresh and ocean waters due to high P input and accompanied eutrophication and hypoxia.

Average P loss due to soil erosion from European croplands is, together with Australia, the smallest of all continents (1.2 and 0.9 kg ha^−1^ yr^−1^ for Europe and Australia, respectively; Table 1). Nevertheless, the overall P losses from agricultural systems in Europe equal losses due to soil erosion (Table 1), and especially Central and Eastern European countries (NEU11) clearly have a negative P balance.

Csatho and Radimszky^62^ in discussing land use and management within the EU argue that the negative P balance and worsening P status in Central- and Eastern European countries is in sharp contrast to past practices in the former EU15 countries, where strong positive P balances and oversupply of P led to environmental and ecological threats. While there is evidence that the level of oversupply in the previous EU15 countries had been falling in the early 1990s due to declining trends in mineral fertilizer use^63^, worsening levels of P undersupply (partly due to the post 1990s rapid decline in fertilizer application) may result in increasingly low yields and in economic and agronomic problems in central and eastern European countries^62^. Even though the central and eastern European countries (NEU11) have lower P loss due to soil erosion compared to the former EU15 (1.2 kg ha^−1^ yr^−1^ versus 2.1 kg ha^−1^ yr^−1^ for the NEU11 compared to EU15, respectively), the overall P balance is nearly balanced in the former EU15 (0.4 kg ha^−1^ yr^−1^) due to considerably higher chemical fertilizer input in the western countries compared to the eastern NEU11 (negative balance thus overall soil depletion of −4.3 kg ha^−1^ yr^−1^). As such, there might be a concern of P deficiency and nutrient depletion in the NEU11 in the future, in spite of comparably lower erosional P losses from soils to waters. With no major geological P deposits in Europe^8^, eastern and western European countries will both be confronted with a harsh political and economic struggle for P fertilizers in the future, with both regions being at the higher end of negative P balances without the addition of chemical fertilizer (−14 and −9.5 kg ha^−1^ yr^−1^ for EU15 and NEU11, respectively; Table 1).

For Asia, China certainly stands out in having extensive programs to save and re-cycle P (e.g., separating human excreta and urine, recovery from sewage sludge, sludge ash and the fertilizer industry^64^). Simultaneously Chinese soils experience the highest chemical fertilizer consumption resulting in a nearly balanced P budget (−0.4 kg ha^−1^ yr^−1^). With having the highest losses of P due to soil erosion by water (12.3 kg ha^−1^ yr^−1^ with only considering rill and interrill erosion, Table 1), a significant reduction in soil erosion rates would contribute tremendously to the national struggle to save P.

Even though P demand is stagnating in some regions (mostly Europe, North America and Australia), today overall demand is nevertheless increasing globally due to population growth, intensification of agriculture and a shift from vegetarian to meat based diet^3,12^. It has been suggested, that a 50% reduction in food and feed waste combined with a 50% reduction in production and consumption of animal products, will allow a 100% conversion to organic agriculture, thus fostering sustainable agriculture and minimizing agricultural production related problems such as greenhouse gas production, biodiversity loss, eutrophication of waters and eco-toxicological related issues^65^. However, the switch to 100% organic production globally would only be possible if rock phosphate was used as a mineral P-fertilizer in organic agriculture with a similar magnitude as it is used today in conventional agriculture^65^.

To conclude, because P supply from geological deposits cannot be increased but phosphorus resources will be increasingly limited in the future globally, reducing soil erosion might be a crucial if not the most important management option to (i) allow decreased fertilizer application and thus save some of the precious P resources today, (ii) stop continuous depletion of eastern European and African soil P storages and (iii) reduce impact to fresh and ocean waters to counteract eutrophication and hypoxia. We would like to point out that RUSLE based erosion rates as the basis to calculate P loss due to erosion only consider rill and inter-rill erosion processes by water, and do not consider erosion processes due to tillage, gullies or landslides. As such, our P loss assessments can be expected to be conservative estimates. Measures to reduce soil erosion will be dependent on region specific characteristics of climate, topography, soils and harvesting aims and will be dependent on economic and topographic feasibility of management options. Adequate and adapted erosion control might be one, or a combination of measures such as (i) no tillage or low tillage, (ii) maintenance of a permanent soil cover achieved by increased vegetation especially cover crops, diversification of plant species, mulching, and/or intercropping, as well as (iii) topography adapted land management (e.g., terracing, strip cropping and contour farming)^66,67^. Especially a combination of sustainable practices could make a serious impact on reducing erosion and the associated P losses in the most vulnerable countries, leading to positive agricultural and environmental outcomes.

## Methods

### RUSLE soil loss

Global soil loss estimates were taken from Borrelli et al.^32^ using the RUSLE-based Global Soil Erosion Modeling platform (GloSEM), a modified large-scale Geographic Information System (GIS) version (RUSLE2015^67^) of the Revised Universal Soil Loss Equation (RUSLE^68^) model. RUSLE belongs to the so called detachment-limited model types where the soil loss (here synonym to RUSLE soil loss^69^) is due to inter-rill and rill erosion processes. The long-term annual soil loss rates (Mg ha^−1^ yr^−1^) refers to the amount of sediment that reaches the end of a specified area (cell) on a hillslope that experiences loss of soil by water erosion. The modeled area does not, in any way, include areas of the slope that experience net deposition over the long term. This is because the displaced soil amount is not routed downslope across each cell from hillslopes to the sink area or the riverine systems through a transport/deposition capacity module. Borrelli et al.^32^ estimated soil loss from croplands as $17_{ - 0.7}^{ + 1}$ Pg yr^−1^ (referring to 1.43 billion ha cropland). For a detailed description of soil loss data used in this study please refer to Borrelli, et al.^32^.

To calculate the loss in soil stock of the topmost 30 cm, bulk density (BD) was taken from the Soilgrids database at 250 m spatial resolution^70^ as an average of the four top soil layers:

$${\mathrm{Soil}}\;{\mathrm{stock}}\;{\mathrm{lost}}\;\left( {\mathrm{\% }} \right) = \frac{{{\mathrm{RUSLE}}\;{\mathrm{soil}}\;{\mathrm{loss}}\;({\mathrm{kg/ha}})}}{{{\mathrm{Soil}}\;{\mathrm{Stock}}\;({\mathrm{kg/ha}})}} * 100$$

### Soil P contents and P loss

Soil P contents were taken from Ringeval, et al.^33^ who combined global data sets from^21,24,36,37,71–74^ (see Supplementary Table 1) in a soil P dynamics model to assess the contributions of the different drivers (P inherited from natural soils, land use and land cover change, soil P input/output due to farming practices, climatic forces, atmospheric deposition, losses though erosion, and soil P buffering capacities, Supplementary Table 1) at the global scale and to simulate the distribution of different P fractions in agricultural soils. The model was run over the period 1900–2005 at a time scale of 1 year. Soil input/output due to farming practices are based on data from Bouwman et al.^21^.

Phosphorus loss from soils (P_loss_) can be induced by (i) erosion/ soil loss or (ii) leaching. We neglected leaching as this can be expected to be of only very minor importance regarding P loss from soils. As such, P_loss_ was defined for each fraction as:

$${\mathrm{P{\scriptsize{loss}}}} = {\mathrm{P{\scriptsize{fraction}}}} *{\mathrm{Soil}}\;{\mathrm{stock}}\;{\mathrm{lost}}\;({\mathrm{\% }})$$

with P_fractions_ in kg ha^−1^yr^−1^ defined according to Ringeval et al.^33^ based on the global distribution of P fractions according to Yang et al.^37^, which summed Hedley fractions^33,37^: labile organic P (organic P extracted with resin and with bicarbonate), stable organic P (extracted with hydroxide), labile inorganic P (inorganic P extracted with resin and bicarbonate), inorganic P bound on secondary minerals (inorganic P extracted with hydroxide), apatite (P extracted with hydrochloric acid), occluded inorganic P (residual P) and total P (as sum of all fractions). Yang et al.^37^ built up a global database of P fractions considering the major USDA soil taxonomy soil orders grouped in three soil weathering categories as slightly, intermediately and highly weathered soils. However, as there is great uncertainty to associate these fractions with functional plant uptake^39,40^ we present (Figs. 3 and 4, Table 1) the total P loss, the plant-available pool as the most labile P which can participate to plant nutrition in a time scale up to months^17^ (i.e., the sum of labile inorganic P, inorganic P bound to secondary minerals, labile and stable organic P) and the very stable pools which would not be plant available at short time scales (i.e., inorganic P associated with minerals such as apatite or occluded P^17^, corresponding to 52% of total soil P in our estimates at the global scale). Losses of plant-available fractions are presented in Fig. 2.

### Modeled area

As the study is based on phosphorus soil content data from Ringeval et al.^33^ we cover the same 1.04 billion ha of global cropland from a total of 1.43 billion ha with a resolution of 0.5° × 0.5° based on the land-use harmonization data of Hurtt et al.^73^. The coverage of the modeled area in Ringeval et al.^33^ was the result of the combination of different datasets with the respective coverages.

### Yearly P fluxes and balance

For atmospheric input data we followed the approach of Ringeval et al.^33^ who based their data set on Wang et al.^36^. Atmospheric P input via dust has also been estimated recently by Aciego et al.^35^ assuming only a tenth (a maximum of 0.1 kg ha^−1^ yr^−1^) of the fluxes estimated by Wang et al.^36^. However, atmospheric fluxes are negligible relative to input from chemical fertilizer or output via erosional fluxes. Nevertheless, we wanted to be sure not to underestimate atmospheric input and thus used the global data set of Wang et al.^36^.

Data for chemical fertilizer, manure, residue input (e.g., plant biomass that remains on/within the soil after the harvest and include root biomass) and plant uptake stems from Ringeval et al.^33^ who originally based their calculations on data from Bouwman et al.^21,75^. Bouwman et al.^21,75^ used data of the International Assessment of Agricultural Knowledge, Science, and Technology for Development^76^ as input for an implementation of the IMAGE model to calculate soil input/output fluxes for the year 2000 corresponding to farming practices (i.e., chemical fertilizer, manure, P in harvest). Residue input and plant uptake used in our study were derived from P in harvest provided by Bouwman et al.^21,75^ following basic assumptions as described in Ringeval et al.^33^. Please note that P de-occlusion (e.g., the flux from occluded to secondary minerals) as a small but continuous replenishment of the soluble P pools was not considered in the approach of Ringeval et al.^33^ but the soil buffering capacity driver was only following sorption and desorption kinetics according to the Langmuir equation with specific parameters taken from Wang et al.^24^. For all input layers to the model of Ringeval et al.^33^ as well as technical information please see Supplementary Table 1 and https://esdac.jrc.ec.europa.eu/themes/global-phosphorus.

We defined the term organic P management as the sum of manure and residue input minus plant uptake following the idea that the sum of these fluxes underlies different regulating mechanisms as atmospheric deposition (diffuse input without regulating management options) or chemical fertilizer (bound to financial, technical and eventually geological availability of minable P).

$${\mathrm{organic}}\;{\mathrm{P}}\;{\mathrm{management}} = {\mathrm{manure}} + {\mathrm{residue}}\;{\mathrm{input}} - {\mathrm{plant}}\;{\mathrm{uptake}}$$

P balance of our systems was calculated with or without chemical fertilizer input as:

$$\begin{array}{l}{\mathrm{Pbalance}} = {\mathrm{atmospheric}}\;{\mathrm{input}} + {\mathrm{organic}}\;{\mathrm{P}}\;{\mathrm{management}}\;\left( + {\mathrm{chemical}}\;{\mathrm{fertilizer}} \right)\\ - {\mathrm{total}}\;{\mathrm{soil}}\;{\mathrm{loss}}\;{\mathrm{due}}\;{\mathrm{to}}\;{\mathrm{soil}}\;{\mathrm{erosion}}\end{array}$$

### Error estimation

For all calculated fluxes and balances errors were estimated as relative, non-symmetric errors to the means in % (e.g., as $x_{ - 6.7\% }^{ + 15.6\% }$)with error propagation using the standard deviation of all P pool data from Ringeval et al.^33^ and the uncertainty of fluxes given for the soil loss data in Borrelli et al.^32^ (Supplementary Fig. 1).

RUSLE is a purely deterministic model in which the product of physical measures is used to derive the amount of soil loss. In such a model, uncertainty in the model output stem from the uncertainty of input factors which then propagate through the model. As such, a sensu stricto assessment of uncertainties of large or global scale modeling is not feasible^77^, because of the unknown uncertainty of all input layers. More specifically, in our approach we do have an estimate of the uncertainty of the layer R (rainfall erosivity) and of the topography related factors (LS factors), but we miss the uncertainty estimates due to the misclassification of land cover (C factor) or the uncertainty associated to the erodibility of soils (K factor). Here, we replicated the approach followed by Borrelli et al.^32^ which estimates uncertainty as a probability distribution using a Bayesian modeling technique. For this application only the cropland areas are considered. The idea is to use the data distribution to estimate the uncertainty of the prediction. The approach used is to approximate the magnitude of the error from the distribution of the values in the layers. Given that RUSLE is based on a product, to simplify, all layers were log-transformed. In other words, each of the input layers was treated as a spatial random field. A random field is a stochastic process defined in terms of expectation and covariance. Once these two parameters are estimated, different simulations for each field can be created. Each simulation has the same parameters, but differs due to the stochasticity of the process. By combining a large number of simulations, the uncertainty is estimated and how it propagates to the model output (soil loss).

For the practical implementation, and as a spatially continuous simulation for each of the layers is impractical, a simulation approach based on Gibbs sampling and an additive model was used. The model is expressed as:

$${\mathrm{z}}\;{\mathrm{(}}S_0{\mathrm{)}} = {\mathrm{z}}\left( R \right) + {\mathrm{z}}\left( {{\mathrm{LS}}} \right) + {\mathrm{z}}\left( K \right) + {\mathrm{z}}\left( C \right) + {\mathrm{e}}\left( s \right)$$

where the z() values are realizations from each of the log-transformed model input layers and e(s) is the spatial component of the model. The additive model uses simulated data derived from the observed instances following a feature distance kernel. For a given observation i, corresponding to the vector of variabes j (j = (S_i_, R_i_, Ls_i_, K_i_, C_i_)), estimates of the mean (µ()_j_) and variance (σ^2^()_j_) of all the variables are derived from the k (k = 100) closest observations in feature space. The simulated vector of j is then drawn from a Gaussian distribution with mean µ()_j_ and variance σ^2^()_j_. The idea is that by selecting similar environmental conditions it is possible to estimate the range variability of the outcome while remaining in the domain of what is physically feasible. The main limitation of this approach is that for numerical stability, several realizations for each simulated point must be used, thus inflating the number of data points. In order to limit the computational burden, the simulated values are then used to fit a Markov Chain Monte Carlo (MCMC) additive model to derive the posterior distribution of each parameter plus the outcome from a random sample of 2×10^6^ observation covering the spatial extent of the layers. The MCMC additive regression model was then used to derive realizations of *z(S*_*0*_*)* (soil loss) from which confidence intervals were calculated. The MCMC model was applied using the JAGS software through the R interface.

Regarding the uncertainty of the P soil pool data, the total soil P (P_TOT_) simulated by Ringeval et al.^33^ is derived from a combination of different datasets describing the main drivers of cycle P in cropland with the soil P dynamic model (see above). The procedure adopted by Ringeval et al.^33^ to estimate how the uncertainty related to each driver propagated to the simulated P_TOT_ can be synthesized as follow. Within a first step, a range of uncertainty for each driver (Supplementary Table 1), defined by bottom and top boundaries, was defined following available information. Since no information about the uncertainty of some drivers (farming practices and soil buffering capacity) were available, an uncertainty of ±30% was arbitrarily assumed. The simulation of P_TOT_ was replicated 30 times. For a given replication, the value of each driver was chosen randomly within the range between the two estimates by assuming a uniform distribution (i.e., assuming that all values between estimates 1 and 2 were equally likely). This was done independently for each grid cell. The 30 replications were used to compute for each grid-cell an average and standard deviation. For more details please see Ringeval et al.^33^.

## Supplementary information

Supplementary Information

Peer Review File

## Data Availability

The data presented in this study (Figs. 1 and 2) as well as all input layers to our modeling approach are directly available at the European Soil Data Centre (ESDAC) of the European Commission – Joint Research Centre: https://esdac.jrc.ec.europa.eu/themes/global-phosphorus. Access to the data is free for all public users.

## References

[1] Bouwman, A. F., Beusen, A. H. W. & Billen, G. Human alteration of the global nitrogen and phosphorus soil balances for the period 1970-2050. *Global Biogeochemical Cycles* **23**, 1-16, 10.1029/2009gb003576 (2009).

[2] Carpenter SR, Bennett EM (2011). Reconsideration of the planetary boundary for phosphorus. Environ. Res. Lett..

[3] Cordell D, Drangert JO, White S (2009). The story of phosphorus: global food security and food for thought. Glob. Environ. Change.

[4] Gilbert N (2009). Environment: the disappearing nutrient. Nature.

[5] Edixhoven JD, Gupta J, Savenije HHG (2014). Recent revisions of phosphate rock reserves and resources: a critique. Earth Syst. Dynam.

[6] Scholz RW, Wellmer FW (2013). Approaching a dynamic view on the availability of mineral resources: what we may learn from the case of phosphorus?. Glob. Environ. Change.

[7] Scholz RW, Wellmer FW (2016). Comment on: “Recent revisions of phosphate rock reserves and resources: a critique” by Edixhoven et al. (2014) - clarifying comments and thoughts on key conceptions, conclusions and interpretation to allow for sustainable action. Earth Syst. Dyn..

[8] Van Kauwenbergh, S. J. World Phosphate Rock Reserves and Resources. Technical Bulletin IFDC-T-75 (2010).

[9] Kohn J, Zimmer D, Leinweber P (2018). Is phosphorus really a scarce resource? Int. J. Environ. Technol. Manag..

[10] Ulrich AE, Frossard E (2014). On the history of a reoccurring concept: phosphorus scarcity. Sci. Total Environ..

[11] MinemakersLimited. Rock Phosphate Price Rockets to US$200/tonne. ASX and Press Release Perth (2008).

[12] Elser J, Bennett E (2011). A broken biogeochemical cycle. Nature.

[13] Amundson R (2015). Soil and human security in the 21st century. Science.

[14] USDA. USDA Economic Research Service (2019).

[15] Quinton JN, Govers G, Van Oost K, Bardgett RD (2010). The impact of agricultural soil erosion on biogeochemical cycling. Nat. Geosci..

[16] Smil V (2000). Phosphorus in the environment: Natural flows and human interferences. Annu. Rev. Energy Environ..

[17] Helfenstein J (2018). Combining spectroscopic and isotopic techniques gives a dynamic view of phosphorus cycling in soil. Nat. Commun..

[18] Riskin SH (2013). The fate of phosphorus fertilizer in Amazon soya bean fields. Philos. Trans. R. Soc. B Biol. Sci.

[19] Frossard E, Condron LM, Oberson A, Sinaj S, Fardeau JC (2000). Processes governing phosphorus availability in temperate soils. J. Environ. Qual..

[20] Sattari SZ, Bouwman AF, Giller KE, van Ittersum MK (2012). Residual soil phosphorus as the missing piece in the global phosphorus crisis puzzle. Proc. Natl Acad. Sci. USA.

[21] Bouwman L (2013). Exploring global changes in nitrogen and phosphorus cycles in agriculture induced by livestock production over the 1900–2050 period. Proc. Natl Acad. Sci. USA.

[22] MacDonald GK, Bennett EM, Potter PA, Ramankutty N (2011). Agronomic phosphorus imbalances across the world’s croplands. Proc. Natl Acad. Sci. USA.

[23] Drangert JO (1998). Fighting the urine blindness to provide more sanitation options. Water SA.

[24] Wang YP, Law RM, Pak B (2010). A global model of carbon, nitrogen and phosphorus cycles for the terrestrial biosphere. Biogeosciences.

[25] MacDonald GK, Bennett EM, Taranu ZE (2012). The influence of time, soil characteristics, and land-use history on soil phosphorus legacies: a global meta-analysis. Glob. Change Biol..

[26] Ockenden MC (2017). Major agricultural changes required to mitigate phosphorus losses under climate change. Nat. Commun..

[27] Liu Y, Villalba G, Ayres RU, Schroder H (2008). Global phosphorus flows and environmental impacts from a consumption perspective. J. Ind. Ecol..

[28] Chen MP, Graedel TE (2016). A half-century of global phosphorus flows, stocks, production, consumption, recycling, and environmental impacts. Glob. Environ. Change.

[29] van Dijk KC, Lesschen JP, Oenema O (2016). Phosphorus flows and balances of the European Union Member States. Sci. Total Environ..

[30] Bennett EM, Carpenter SR, Caraco NF (2001). Human impact on erodable phosphorus and eutrophication: a global perspective. Bioscience.

[31] Pimentel D (2006). Soil erosion: a food and environmental threat. Environ. Dev. Sustainability.

[32] Borrelli P (2017). An assessment of the global impact of 21st century land use change on soil erosion. Nat. Commun..

[33] Ringeval B (2017). Phosphorus in agricultural soils: drivers of its distribution at the global scale. Global Change Biol.

[34] Liu, Y. The Human Intensified Global Phosphorus Flows and Environmental Impacts. Interim Report. IR-06-081, International Institute for Applied Systems Analysis, Laxenburg, Austria, 1-30 (2006).

[35] Aciego SM (2017). Dust outpaces bedrock in nutrient supply to montane forest ecosystems. Nat. Commun..

[36] Wang R (2015). Significant contribution of combustion-related emiscions to the atmospheric phosphorus budget. Nat. Geosci..

[37] Yang X, Post WM, Thornton PE, Jain A (2013). The distribution of soil phosphorus for global biogeochemical modeling. Biogeosciences.

[38] Hedley MJ, Stewart JWB, Chauhan BS (1982). Changes in inorganic and organic soil-phosphorus fractions induced by cultivation practices and by laboratory incubations. Soil Sci. Soc. Am. J..

[39] Tiessen H, Stewart JWB, Cole CV (1984). Pathways of phosphorus transformations in soils of differing pedogenesis. Soil Sci. Soc. Am. J..

[40] Zehetner F, Wuenscher R, Peticzka R, Unterfrauner H (2018). Correlation of extractable soil phosphorus (P) with plant P uptake: 14 extraction methods applied to 50 agricultural soils from Central Europe. Plant Soil Environ..

[41] Helfenstein J (2020). Estimates of mean residence times of phosphorus in commonly considered inorganic soil phosphorus pools. Biogeosciences.

[42] Goudie, A. Encyclopedia of Geomorphology (Taylor & Francis, 2013).

[43] Lugato E (2018). Soil erosion is unlikely to drive a future carbon sink in Europe. Sci. Adv..

[44] Lal R (2003). Soil erosion and the global carbon budget. Environ. Int..

[45] Beusen AHW, Dekkers ALM, Bouwman AF, Ludwig W, Harrison J (2005). Estimation of global river transport of sediments and associated particulate C, N, and P. Glob. Biogeochem. Cycles.

[46] Kronvang B, Vagstad N, Behrendt H, Bogestrand J, Larsen SE (2007). Phosphorus losses at the catchment scale within Europe: an overview. Soil Use Manag..

[47] Stackpoole SM, Stets EG, Sprague LA (2019). Variable impacts of contemporary versus legacy agricultural phosphorus on US river water quality. Proc. Natl Acad. Sci. USA.

[48] Baker DB (2014). Phosphorus loading to Lake Erie from the Maumee, Sandusky and Cuyahoga rivers: the importance of bioavailability. J. Gt. Lakes Res..

[49] Wang X (2011). Estimating non-point source pollutant loads for the large-scale basin of the Yangtze River in China. Environ. Earth Sci..

[50] Stoorvogel JJ, Smaling EMA, Janssen BH (1993). Calculating soil nutrient balances in africa at different scales .1. Supra-national scale. Fertilizer Res..

[51] Borbor-Cordova MJ, Boyer EW, McDowell WH, Hall CA (2006). Nitrogen and phosphorus budgets for a tropical watershed impacted by agricultural land use: Guayas, Ecuador. Biogeochemistry.

[52] Panagos P (2017). Global rainfall erosivity assessment based on high-temporal resolution rainfall records. Sci. Rep..

[53] Buresh, R. J., Smithson, P. C. & Hellums, D. T. In *Replenishing Soil Fertility in Africa SSSA Special Publication* 111–149 (Soil Science Society of America and American Society of Agronomy, 1997).

[54] Manyong VM, Makinde KO, Sanginga N, Vanlauwe B, Diels J (2001). Fertiliser use and definition of farmer domains for impact-oriented research in the northern Guinea savanna of Nigeria. Nutr. Cycl. Agroecosyst..

[55] Pypers P (2012). Combining mineral fertilizer and green manure for increased, profitable cassava production. Agron. J..

[56] Chikowo R (2010). Nitrogen and phosphorus capture and recovery efficiencies, and crop responses to a range of soil fertility management strategies in sub-Saharan Africa. Nutr. Cycl. Agroecosyst..

[57] Sanchez PA (2010). Tripling crop yields in tropical africa. Nat. Geosci..

[58] Tully K, Sullivan C, Weil R, Sanchez P (2015). The state of soil degradation in Sub-Saharan Africa: baselines, trajectories, and solutions. Sustainability.

[59] Omuto, C. T. & Vargas, R. R. Soil nutrient loss assessment in Malawi. Technical Report. FAO, UNEP and UNDP, 1–64 (2018).

[60] Franke AC, van den Brand GJ, Vanlauwe B, Giller KE (2018). Sustainable intensification through rotations with grain legumes in Sub-Saharan Africa: a review. Agriculture Ecosyst. Environ..

[61] Prestele R, Hirsch AL, Davin EL, Seneviratne SI, Verburg PH (2018). A spatially explicit representation of conservation agriculture for application in global change studies. Global Change Biol..

[62] Csatho P, Radimszky L (2009). Two worlds within EU27: sharp contrasts in organic and mineral nitrogen-phosphorus use, nitrogen-phosphorus balances, and soil phosphorus status: widening and deepening gap between Western and Central Europe. Commun. Soil Sci. Plant Anal..

[63] Ott C, Rechberger H (2012). The European phosphorus balance. Resour., Conserv. Recycling.

[64] Zhou K, Barjenbruch M, Kabbe C, Inial G, Remy C (2016). Phosphorus recovery from municipal and fertilizer wastewater: China’s potential and perspective. J. Environ. Sci.

[65] Muller A (2017). Strategies for feeding the world more sustainably with organic agriculture. Nat. Commun..

[66] Panagos P (2015). Estimating the soil erosion cover-management factor at the European scale. Land Use Policy.

[67] Panagos P (2015). The new assessment of soil loss by water erosion in Europe. Environ. Sci. Policy.

[68] Renard, K., Foster, G., Weesies, G., McCool, D. & Yoder, D. Predicting soil erosion by water: a guide to conservation planning with the Revised Universal Soil Loss Equation (RUSLE). Vol. 703 (Department of Agriculture, Agricultural Research Service, 1997).

[69] Nearing MA, Yin SQ, Borrelli P, Polyakov VO (2017). Rainfall erosivity: an historical review. Catena.

[70] ISRIC. World Soil Information SoilGrids – an automated system for global soil mapping. Available for download at https://soilgrids.org (2016).

[71] Van Oost K (2007). The impact of agricultural soil erosion on the global carbon cycle. Science.

[72] Decharme B, Martin E, Faroux S (2013). Reconciling soil thermal and hydrological lower boundary conditions in land surface models. J. Geophys. Res..

[73] Hurtt GC (2011). Harmonization of land-use scenarios for the period 1500-2100: 600 years of global gridded annual land-use transitions, wood harvest, and resulting secondary lands. Climatic Change.

[74] Krinner G (2005). A dynamic global vegetation model for studies of the coupled atmosphere-biosphere system. Global Biogeochem. Cycles.

[75] Bouwman A, Kram T, Klein Goldewijk K (2006). Integrated modelling of global environmental change. Overv. IMAGE.

[76] McIntyre, B. D., Herren, H. R., Wakhungu, J. & Watson, R. T. International Assessment of Agricultural Knowledge, Science and Technology for Development (IAASTD), Global Report. 590 (Island Press. Washington, DC, 2009).

[77] Auerswald K, Kainz M, Fiener P (2003). Soil erosion potential of organic versus conventional farming evaluated by USLE modelling of cropping statistics for agricultural districts in Bavaria. Soil Use Manag..

[78] Meybeck M (1982). Carbon, nitrogen, and phosphorus transport by world rivers. Am. J. Sci..

[79] Smil V (1999). Nitrogen in crop production: an account of global flows. Glob. Biogeochem. Cycles.

[80] Mackenzie FT, Vera LM, Lerman A (2002). Century-scale nitrogen and phosphorus controls of the carbon cycle. Chem. Geol..

[81] Koci J (2020). Effect of reduced grazing pressure on sediment and nutrient yields in savanna rangeland streams draining to the Great Barrier Reef. J. Hydrol..

