# Supplementary material for: Global phosphorus shortage will be aggravated by soil erosion
Source: Nat Commun. 2020 Sep 11;11:4546. doi: 10.1038/s41467-020-18326-7 (PMC7486398; doi:10.1038/s41467-020-18326-7)
Supplement: Supplementary file 1 — Supplementary Information [file 41467_2020_18326_MOESM1_ESM.pdf]

Supplementary Information for

Global Phosphorus shortage will be aggravated by soil erosion

by

Christine Alewell et al.

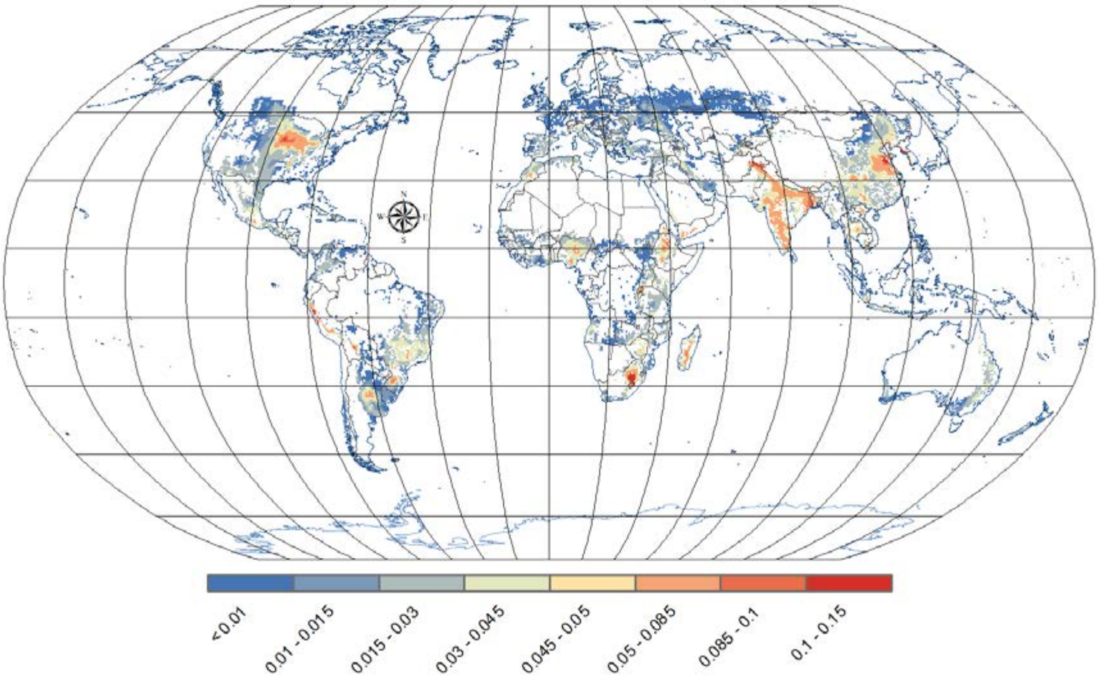

Supplementary Figure 1. Uncertainty of the global average phosphorus (P) losses due to soil erosion presented as the standard deviation of the Markov Chain Monte Carlo (MCMC) simulated values.

Supplementary Table 1: Description of the drivers of the P dynamics model applied by Ringeval, et al. [1] and the two estimates used to assess the uncertainty of the P pool data associated with each driver

Driver name	Description	Description of the two estimates used to assess the uncertainty associated with the driver and data sets used
BIOG<br>(natural soil biogeochemical background)	P inherited from natural soils at the time of conversion to agriculture. P in natural soils was also used to approach P in agricultural soils at the beginning of the simulation (in 1700, initial conditions).	Use of the mean and standard deviation associated to P_{TOT} provided by Yang, et al. [2]:<br>BIOG1 = $P_{TOT} - \sqrt{3}.\ std_{PTOT}$<br>BIOG2 = $P_{TOT} + \sqrt{3}.\ std_{PTOT}$<br>Only the uncertainty associated with P_{TOT} was accounted for (i.e. the same contribution of the different forms to P_{TOT} was used in BIOG1 and BIOG2).
LUCC<br>(land use and land cover change)	Decrease/increase of agricultural soil P resulting from land conversion within the same grid cell	Uncertainty not taken into account. Land Use Harmonization data set Hurtt, et al. [3]
FARM<br>(farming practices)	Soil P input/output corresponding to farming practices	We made the soil budget (FARM input – output) vary around the value provided by Bouwman, et al. [4].<br>FARM1 = 70% of the soil budget computed<br>FARM2 = 130% of the soil budget computed<br>The same contribution of the different fluxes (chemical fertilizer, manure, residue, uptake) to the soil budget was used in FARM1 and FARM2.
CLIM<br>(soil temperature and soil water content)	Effect of soil temperature and soil water content on P weathering and P mineralization	Use of the simulations provided by 2 different DGVMs<br>CLIM1 = soil water content and temperature simulated by ISBA Decharme, et al. [5]<br>CLIM2 = soil water content and temperature simulated by ORCHIDEE Krinner, et al. [6]
DEPO<br>(atmospheric deposition)	Soil P input resulting from deposition of atmospheric P	Use of the mean and upper/lower boundaries provided in Wang, et al. [7]<br>DEPO1=25% of mean deposition provided; DEPO2=175 % of mean deposition provided<br>The uncertainty concerns the total deposition.
BUFF<br>(soil buffering capacity)	Soil properties describing the replenishment of the inorganic labile P pool and to adsorb labile P on its particles	BUFF1 = 70% of S_{max} provided by Wang, et al. [7]<br>BUFF2 = 130% of S_{max} provided by Wang, et al. [7]<br>We considered K_s and S_{max} as co-varying and described both BUFF1 and BUFF2 by the same K_S/S_{max} ratio as the one provided by Wang, et al. [7].
LOSS (P losses through erosion)	Losses due to water erosion and run-off processes	Use of two 'scenarios' about flux of eroded sediment (fsediment) provided by Van Oost, et al. [8]<br>LOSS1 = computation of losses using the lower “normal” scenario provided by Van Oost, et al. [8];<br>LOSS2 = computation of losses using the higher scenario provided by Van Oost et al. [2007]<br>(fsediment in LOSS2 = fsediment in LOSS1*1.26)

1 Ringeval, B. *et al.* Phosphorus in agricultural soils: drivers of its distribution at the global scale. *Global Change Biology*, n/a-n/a, doi:10.1111/gcb.13618 (2017).
2 Yang, X., Post, W. M., Thornton, P. E. & Jain, A. The distribution of soil phosphorus for global biogeochemical modeling. *Biogeosciences* **10**, 2525-2537, doi:10.5194/bg-10-2525-2013 (2013).
3 Hurtt, G. C. *et al.* Harmonization of land-use scenarios for the period 1500-2100: 600 years of global gridded annual land-use transitions, wood harvest, and resulting secondary lands. *Climatic Change* **109**, 117-161, doi:10.1007/s10584-011-0153-2 (2011).
4 Bouwman, L. *et al.* Exploring global changes in nitrogen and phosphorus cycles in agriculture induced by livestock production over the 1900–2050 period. *Proceedings of the National Academy of Sciences* **110**, 20882-20887 (2013).
5 Decharme, B., Martin, E. & Faroux, S. Reconciling soil thermal and hydrological lower boundary conditions in land surface models. *Journal of Geophysical Research-Atmospheres* **118**, 7819-7834, doi:10.1002/jgrd.50631 (2013).
6 Krinner, G. *et al.* A dynamic global vegetation model for studies of the coupled atmosphere-biosphere system. *Global Biogeochemical Cycles* **19**, doi:10.1029/2003gb002199 (2005).
7 Wang, R. *et al.* Significant contribution of combustion-related emiscions to the atmospheric phosphorus budget. *Nature Geoscience* **8**, 48-54, doi:10.1038/ngeo2324 (2015).
8 Van Oost, K. *et al.* The Impact of Agricultural Soil Erosion on the Global Carbon Cycle. *Science* **318**, 626-629 (2007).
