# Supplementary material for: Global phosphorus shortage will be aggravated by soil erosion
Source: Nat Commun. 2020 Sep 11;11:4546. doi: 10.1038/s41467-020-18326-7 (PMC7486398; doi:10.1038/s41467-020-18326-7)
Supplement: Supplementary file 2 — Peer Review File [file 41467_2020_18326_MOESM2_ESM.docx]

Peer Review Information

**Journal:** Nature Communications

**Manuscript title:** Global phosphorus shortage will be aggravated by soil erosion

**Corresponding author name(s):** Prof Christine Alewell

**Editorial notes:**

**Transferred manuscripts**

This manuscript has been previously reviewed at another journal that is not operating a transparent peer review scheme. This document only contains reviewer comments and rebuttal letters for versions considered at *Nature Communications*.

**Transferred manuscripts (no peer review at *Nature Communications*)**

This manuscript has been previously reviewed at another journal that is not operating a transparent peer review scheme. The manuscript was considered suitable for publication without further review at *Nature Communications*.

**Redactions – transferred manuscripts (mention of previous referee reports from elsewhere)**

This manuscript has been previously reviewed at another journal that is not operating a transparent peer review scheme. This document only contains reviewer comments and rebuttal letters for versions considered at *Nature Communications*. Mentions of prior referee reports have been redacted.

**Redactions – transferred manuscripts (mention of the other journal)**

This manuscript has been previously reviewed at another journal that is not operating a transparent peer review scheme. This document only contains reviewer comments and rebuttal letters for versions considered at *Nature Communications*. Mentions of the other journal have been redacted.

**Redactions – unpublished data**

Parts of this Peer Review File have been redacted as indicated to maintain the confidentiality of unpublished data.

**Redactions – confidential patient information**

Parts of this Peer Review File have been redacted as indicated to maintain patient confidentiality.

**Redactions – published data**

Parts of this Peer Review File have been redacted as indicated to remove third-party material where no permission to publish could be obtained.

**Amendments – published data**

[Figure X/The figure on page X] in this Peer Review File has been amended to remove third-party material where no permission to publish could be obtained.

**Published data (credit line for image)**

[Figure X/The figure on page X] in this Peer Review File is reproduced with permission from [Copyright Holder].

**Redactions – reviewer opt-out**

Parts of this Peer Review File have been redacted as indicated as we could not obtain permission to publish the reports of reviewer ###.

**Reviewer comments in marked-up manuscript**

In their review of the [first/second/third/...] version of this manuscript, reviewer ### added their comments to the manuscript file. These comments, excluding minor textual revisions, have been copied into this Peer Review File.

**Reviewer comments & decisions:**

**Reviewer comments, first version:**

Reviewers' comments:

Reviewer #1 (Remarks to the Author):

This paper offers novel estimates of P loss via erosion at the global scale, including spatial heterogeneity. I think this is a useful contribution to the discussion on global P dynamics. However, the underlying data are poorly described in the present paper, I have reservations about attempting to model multiple soil P fractions at this scale (and further, I question whether that is even entirely necessary), and discussion concerning management effects on soil erosion is largely absent. I offer more detailed feedback below.

Title – spell out Phosphorus

Throughout manuscript – the correct spelling is phosphorus. See:
https://www.nature.com/articles/426119c

The manuscript requires substantial English-language editing. I will not dwell on this in my review, but I recommend that the authors and editors work to improve English grammar throughout in future versions of the paper.

Introduction
Lines 25-26 – “no biotic mobilization of P” – this statement seems too sweeping to me. Biological organisms, including humans, do several things to access P. I suggest editing this sentence to emphasize the point that there is no analog of N2 fixation in the P cycle.

Starting on Line 45 – I think this is a compelling argument for the value of this study. However, many studies do include estimates of P transport in streams and rivers out of watersheds, which should inherently capture erosion-related P losses. I suggest the authors consider this, and adjust their argument to emphasize that while P losses via rivers have been modeled, the contribution of soil erosion in particular remains poorly characterized (including spatial heterogeneity). This point is in the current manuscript, but it could be made more clearly.

Line 123 – Can the authors provide more nuance on the “expected shortage of P supply from industrial fertilizers in the future”? There is disagreement in the literature concerning the timeline of this resource dilemma, so I think it is important to describe it using the best information available, highlight the significant concern it poses, as well is the uncertainty surrounding the timeline.

Line 136 – The fact that only on-site displacement of soils is considered (and not net catchment output) is concerning to me. The authors mention that displaced soil might be re-located to other parts of the fields or buried in depositional places, but then go on to state that “the considered on-site field management will clearly be confronted with substantial P losses due to soil erosion by water.” This is not clear to me. I suggest that the authors elaborate on this point, citing any relevant literature. Furthermore, the sensitivity of the results to this assumption is worth considering more carefully.

Line 231 – Can the authors offer specific suggestions for methods to decrease soil erosion? That would be more valuable than a general call to limit soil erosion, which we can all agree on already.

General comment – only the top 30 cm of soil is considered. What about deeper soils that become part of the plow layer over time as surface soils erode? Given the long time horizons considered in this study, it seems to me that this could have implications for whether the loss of P in eroded soils necessarily leads to less P available to crops.

Line 288 – why use the term “plant management” instead of including separate terms for manure, plant residues, and crop harvests?

Line 279 – where did Bouwman et al. obtain the data? It would be good to know how the data were originally generated originally without having to trace this back through other papers.

Line 263 – similarly to my previous comment, I think the authors need to provide more information on the origin of the soil phosphorus data. As written, the reader is required to read multiple other papers to understand the origin of the key data in the current paper. This paper should be able to stand on its own more. It seems unlikely to me that Hedley fractionation data for the spatial scale used in this study has firm empirical grounding. Furthermore, do these soil P estimates take into account the build-up of legacy P over time in cropland soils?

Line 290 – Error estimation – I commend the authors for including this section in their study. Too many manuscripts of this kind do not do so.

The quality of Figure 3 needs to be improved. Again, I am skeptical of the individual P fractions included here for the global scale.

General comment – there needs to be more discussion in how soil management can impact soil erosion (for better or worse).


Reviewer #2 (Remarks to the Author):

The manuscript entitled ‘P shortage will be aggravated by soil erosion in Europe, Africa, and South America’ pairs two of the co-authors’ datasets on erosion rates and soil P content to create a novel dataset of losses of P through erosion of cropland globally. This is paired with other existing data on agricultural P flows in order to characterize the importance of soil erosion as a loss pathway (which affects both food security and water quality). A spatially explicit dataset of global erosion losses is absolutely an important contribution to the field. As noted by the authors, there are a diversity of global estimates that agree that it is an important loss, but none that really give global coverage while also accounting for smaller spatial scale variations in controlling factors. The article is well written, but I believe a few important pieces of information and contextual comparisons are missing to ensure that all readers can easily and accurately use the results of this study. I realize that one of the biggest challenges with this type of piece can be the word count limit and thus decisions have to be made on what to present (and also at time on the scope of the work). At the same time however, with how high impact some of these global pieces can get, it seems crucial that important findings be properly framed. As such, I support this article being published after these changes have been made (barring any red flags of course from other reviewers with different areas of expertise).

I believe there are 4 areas that warrant a little more nuance and attention (and I apologize in advance if some of this is in the SI material that might not have made it to me. I only saw an SI Figure and not SI text of tables). I have included some line comments in the attached PDF to support these points.

1) Explicitly mention the reference year(s) for the databases used and then what is the target year for the numbers given for this analysis.
2) In relation to the above, these high loss numbers from erosion (which are quite startling and thus why this paper is likely to be high impact) could be better contextualized with regards to soil P storage, both in terms of legacy P that can be used (or lost) in the future (e.g., Sattari et al. 2012 PNAS, McCrackin et al. 2018 BGC) and also sequestration (e.g., Riskin et al. 2013 philo transactions royal society B). There could also be slightly more nuanced consideration of human and animal excreta recycling, as well as total losses of P to water vs the erosion rates described here.
3) Make datasets, interpretations, and figures easily accessible to all. I was missing links to the full spatially explicit datasets, didn’t see an analysis of uncertainty, and some of the figures and the captions could be clearer (including to color blind people).


Reviewer #3 (Remarks to the Author):

The objective of the study, to determine spatially distributed P losses from soil erosion for the world, is important and well justified. The approach is based on assumptions and estimates from previous studies that have not previously been combined at this spatial scale, therefore, the novelty is found in the fact these authors are making P loss estimates at a resolution of 250 m. This allows for a more detailed discussion of regions of the world that are experiencing excessive P losses and how this may relate to the sustainability of our P fertilizer reserves. The paper has potential to make a sound contribution to scientific literature and thinking.

However, it appears the authors made minor efforts to validate the estimates of their approach. They made a very broad comparison of their estimates with Kronvang et al. (2007). In lines 186 to 196 they concluded that their estimates of 1.2 kg P/ha loss from soil erosion in Europe was on the same order of magnitude with estimates presented by Kronvang et al (2007), which were 0.05 to 3 kg P/ha/yr. However, a closer look at data from Kronvang et al. (2007) shows that the range of erosion estimates was highly skewed by one of the 17 watersheds. The range of P loss from the remaining 16 watersheds was only 0.05 to 1 kg P/ha and the median (for all 17) was 0.41 kg P/ha. Therefore, their estimate of 1.2 kg P/ha is nearly three times greater than the median from the paper they cite.

Furthermore, there is a wealth of other published data that they could use to validate the estimates from their model. Stackpoole et al. (2019) estimated P balance, including P loss in surface water, for 143 watersheds in the United States. Figure S1 from their paper shows that 75% of all watersheds had less than 1 kg P/ha lost through surface water (which does not account for point sources). Data used by Stackpoole et al. (2019) is available in a published dataset by De Cicco et al. (2017). Baker et al. (2014) determined P losses from three major watersheds draining into lake Erie in North America and, after subtracting out P from point-source discharges, the P export in river due to non-point source was between 1 and 1.3 kg P/ha/yr. The authors estimate that P loss due to erosion in North America is 4.6 kg P/ha/yr, which is over 4 times the estimates from above cited sources of measured data.

It is important to note that the authors state their model does not calculate the net catchment output, but rather on-site displacement of soils (lines 136-138), however, they should be able to use published information about catchment delivery ratios to determine if their estimates are valid relative to data on published catchment-scale P losses. I recommend the authors present additional data related to validation of estimates produced by their modeling approach compared to available measured data.

Editorial comments

Line 65-66: Please note that Asia also has a positive balance in Table 1.

Table 1: correct spelling of “Amerika” to “America”

References

Baker, D.B., R. Confesor, D.E. Ewing, L.T. Johnson, J.W. Kramer, and B.J. Merryfield. 2014. Phosphorus Loading to Lake Erie from the Maumee, Sandusky, and Cuyahoga rivers: the importance of bioavilability. J. Great Lakes Res. 40:502-517. http://dx.doi.org/10.1016/j.jglr.2014.05.001

De Cicco, L.A., Sprague, L.A., Murphy, J.C., Riskin, M.L., Falcone, J.A., Stets, E.G., Oelsner, G.P., and Johnson, H.M., 2017, Water-quality and streamflow datasets used in the Weighted Regressions on Time, Discharge, and Season (WRTDS) models to determine trends in the Nation’s rivers and streams, 1972-2012 (ver. 1.1 July 7, 2017): U.S. Geological Survey data release, https://doi.org/10.5066/F7KW5D4H.

Stackpoole, S.M, E.G. Stets, and L.A. Sprague. 2019. Variable impacts of contemporary versus legacy agricultural phosphorus on US river water quality. Proc. Natl. Acad. Sci. 116:20562-20567. www.pnas.org/cgi/doi/10.1073/pnas.1903226116

**Author rebuttal, first version:**

Answer to Reviewer #1:

*1. This paper offers novel estimates of P loss via erosion at the global scale, including spatial heterogeneity. I think this is a useful contribution to the discussion on global P dynamics. However, the underlying data are poorly described in the present paper, I have reservations about attempting to model multiple soil P fractions at this scale (and further, I question whether that is even entirely necessary), and discussion concerning management effects on soil erosion is largely absent. I offer more detailed feedback below.*

We describe the origin of the underlying data in more detail in the manuscript (please see our answer to your comment 12.). We fully agree with the Reviewer that considering Hedley fraction at the global scale is difficult but as only a small fraction of total P is available to the plants, considering different fractions in soil P is required to assess significance of P loss. It is particularly relevant in a long-term perspective to assess how P can limit yield in croplands. Orthophosphate ions (H2PO4^-^ and HPO4^2-^) in the soil solution constitute the main form of P taken up by the plants roots. At equilibrium, soil water contains only about 1% of the P required to sustain normal plant growth for a season. Thus, phosphates removed by plant and microbial uptake must be continually replenished from the inorganic, organic and microbial P pools in the soil (Liu et al., 2008). At field scale, bioavailable P is commonly defined as the sum of P in solution and P that could be replenished into a growing season.

P available to plants is usually assessed through chemical extractions despite the difficulties to relate them to plant uptake (see e.g. (Tiessen et al., 1984;Zehetner et al., 2018)). Many chemical extractions have been used throughout the World: e.g., Olsen P, Dyer P and others, but the Hedley fractionation has some advantages as compared to other methods. The Hedley method fractionates soil P into different forms of P that are removed sequentially with successively stronger extraction agents. These fractions are usually merged into various inorganic and organic pools and classified according to their plant availability, which gives a comprehensive picture of the different forms of P in soils (Table S1). In addition, to our knowledge, (Yang et al., 2013) is the only published database providing information about the global

Page 1/9

University of Basel Environmental Geosciences Bernoullistrasse 30 4056 Basel, Switzerland [umweltgeo.unibas.ch](http://umweltgeo.unibas.ch)

Christine Alewell

T +41 61 207 04 77

[Christine.alewell@unibas.ch](mailto:Christine.alewell@unibas.ch)

distribution of P in unmanaged soils and it was based on the concepts developed in the Hedley method. In our point of view, Hedley fractionation based pools are also better than considering two

conceptual pools as used in (Sattari et al., 2012), because it is more process-based. Work is in progress to improve our representation of soil P pools based in Hedley pools at the global scale (see for instance, (Helfenstein et al., 2020)). However, as we are aware of the uncertainties, we do not give detailed spatially discrete data for P fractions (Fig. 2 and available data is only on total soil P) but only average estimates for continents and regions (Figure 3 and Table 1). For the global map of total P displacement (Fig. 2) we only considered the fractions which are commonly called labile and stabile organic and inorganic P but did not consider P associated with apatite or occluded P, as these mineral associated fractions were traditionally considered the “residual fractions” which are not plant available. However, there is convincing evidence (e.g., (Riskin et al., 2013;Sattari et al., 2012)) that these fractions are likely to be plant available once the readily available pools are depleted. Thus, we included these “residual” fractions in Table 1 and Figures 3. We added a paragraph on the consequences of consideration versus non-consideration of these mineral associated P fractions (lines 164-184).

Regarding the discussion of the management effects, we included possible management options on how to reduce soil erosion (please see lines 347-355). However, we would like to state that soil loss occurs not because of any lack of knowledge on how to protect soils, but a lack in policy governance. The lack in policy governance is due to a lack in research, knowledge and dissemination of the spatial distribution of soil erosion and sediment source attribution from water and wind erosion. Our study, aims to address these latter issues.

2. *Title – spell out Phosphorus*

*Throughout manuscript – the correct spelling is phosphorus. See:*

[*https://www.nature.com/articles/426119c*](https://www.nature.com/articles/426119c)

Sorry for this mistake, we changed it throughout the manuscript.

0. *The manuscript requires substantial English-language editing. I will not dwell on this in my review, but I recommend that the authors and editors work to improve English grammar throughout in future versions of the paper.*

The manuscript was language edited by one of our native speaker co-authors, David Robinson. We do hope that it thus meets the standards of Nature Communications. Please let us know if you need us to engage a professional proofreader.

1. *Introduction*

*Lines 25-26 – “no biotic mobilization of P” – this statement seems too sweeping to me. Biological*

*organisms, including humans, do several things to access P. I suggest editing this sentence to emphasize*

*the point that there is no analog of N2 fixation in the P cycle.*

Thanks for pointing this out, sentence changed (lines 27-30)

2. *Starting on Line 45 – I think this is a compelling argument for the value of this study. However, many studies do include estimates of P transport in streams and rivers out of watersheds, which should inherently capture erosion-related P losses. I suggest the authors consider this, and adjust their argument to emphasize that while P losses via rivers have been modeled, the contribution of soil erosion in particular remains poorly characterized (including spatial heterogeneity). This point is in the current manuscript, but it could be made more clearly.*

While it is not easy to find large-scale studies with data on source area related P river loads, we did find some studies to relate river P exports to source area (e.g. assessing kg ha^-1^yr^-1^; (Baker et al., 2014;Beusen et al., 2005;Borbor-Cordova et al., 2006;Koci et al., 2020;Kronvang et al., 2007;Stackpoole et al., 2019;Stoorvogel et al., 1993;Wang et al., 2011)). However, to validate our on-site P soil loss

Page 2/9

approach with off-site P river export data requires two prerequisites: (i) an estimation of the agricultural erosion and runoff contribution to total P loads in the rivers and (ii) an assumption on sediment delivery rates to estimate P loads to rivers from on-site P soil loss. In following these assumptions, we could compare global values of Beussen et al. 2005 as well as large scale regional studies for each continent with the exception of Oceania (please see lines 189-238 including Table 3). The comparison shows, that we are well in the range of the off-site assessments with rather a slight underestimation (which, however could be partly due to the assumed range in sediment delivery rates of 11-30%, which we took from recent large scale studies (Goudie, 2013;Lal, 2003;Lugato et al., 2018)).

6. *Line 123 – Can the authors provide more nuance on the “expected shortage of P supply from industrial fertilizers in the future”? There is disagreement in the literature concerning the timeline of this resource dilemma, so I think it is important to describe it using the best information available, highlight the significant concern it poses, as well is the uncertainty surrounding the timeline.*

We do think this is a highly interesting discussion. Recent literature is controversial whether P supply from rock reserves of future decades will be a real physical scarcity or will be limited by economic and technical constraints. As we have been encouraged by the editor to expand our manuscript, we added a paragraph on this interesting debate (please see lines 37-53). However, the only definite conclusion, which is possible to draw at today’s state of knowledge, is, that future P supply will be scarce for those countries which have no claim on the remaining major geological P deposits. The reasons for this scarcity might be physical, technical or economic constraints.

7. *Line 136 – The fact that only on-site displacement of soils is considered (and not net catchment output) is concerning to me. The authors mention that displaced soil might be re-located to other parts of the fields or buried in depositional places, but then go on to state that “the considered on-site field management will clearly be confronted with substantial P losses due to soil erosion by water.” This is not clear to me. I suggest that the authors elaborate on this point, citing any relevant literature. Furthermore, the sensitivity of the results to this assumption is worth considering more carefully.*

Large scale assessments estimate sediment delivery rates (e.g. the percent of sediment eroded which are carried to the water bodies) between 2 -30% (Goudie, 2013;Lal, 2003;Lugato et al., 2018). Of course, the transport of P to freshwaters is connected to increases in eutrophication of rivers, streams, lakes and oceans, which induces many highly detrimental off-site effects such as biodiversity loss, algae blooms, loss in fisheries and many more, finally yet importantly, damage to infrastructure. However, the on-site loss of phosphorus due to soil erosion is often neglected, probably partly because most studies originate in the industrialised world with sufficient supplies of mineral P fertilizer to replenish soil pools (no economic P scarcity). We would like to point out, that even if 70 - 90% of the P lost from agricultural soils might be re-deposited within catchments, potential threats of P loss from soils are not reduced. Erosion (and thus P loss) occurs predominantly on agricultural soils while re-deposition will mostly occur in depositional hollows, wetlands, riparian zones or buffer strips. Thus, P is lost as a nutrient on food and feed production sites but re-deposited as a potential ecological threat to biodiversity and ecosystem health due to its eutrophication effect in less intensively or unmanaged ecosystems. We included this discussion in the manuscript (please see lines 199-204). Furthermore, this study explicitly addresses the future perspective, when the world will be ruled by an increased P fertilizer scarcity, be it for physical, economic or technical reasons. As stated above, we also included a comparison to river P loads to “validate” our assessment with the catchment output perspective (lines 189-238).

8. *Line 231 – Can the authors offer specific suggestions for methods to decrease soil erosion? That would be more valuable than a general call to limit soil erosion, which we can all agree on already.*

We included a short paragraph on possible management strategies and measures (lines 347-355). However, we would like to state that soil loss occurs not because of any lack of knowledge on how to

Page 3/9

protect soils, but a lack in policy governance. The lack in policy governance is due to a lack in research, knowledge and dissemination on spatial distribution of soil erosion and sediment source attribution from water and wind erosion. Our study, in line with many other recent spatially distributed large-scale studies, aims to address these latter issues. As such, we do feel there is no room in this manuscript for an in-depth discussion on measures to reduce soil erosion.

9. *General comment – only the top 30 cm of soil is considered. What about deeper soils that become part of the plow layer over time as surface soils erode? Given the long time horizons considered in this study, it seems to me that this could have implications for whether the loss of P in eroded soils necessarily leads to less P available to crops.*

The original data set of (Ringeval et al., 2017) targeted the plough layer, as these layers are of most interest for plant nutrition and thus food and feed production. In considering the upper 30 cm of soils, and using the global average soil erosion rates of (Borrelli et al., 2017) of 2.8 t ha^-1^yr^-1^ (with an assumed rough but realistic bulk density of 1 g cm^-3^ for loosely structured A horizons), 30 cm would be eroded in ~1000 years (even with hot spots of erosion of 30 t ha^-1^yr^-1^, 30 cm would only be eroded in 100 years). Furthermore, and even though highly interesting, this discussion is a bit arbitrary. With continuing P supply due to fertilization, deeper soil horizons will slowly be levelling to similar P concentrations as surface soils today, as they are more and more exposed to P fertilizer and ploughing (as has already happened over the last thousands of years, very since humankind started agricultural land management and thus considerably increased erosion rates). Thus, we think this topic is out of the scope of this paper but might be addressed in sub-sequent studies focusing on the “soil depth, erosion processes and long term effects”.

10. *Line 288 – why use the term “plant management” instead of including separate terms for manure, plant residues, and crop harvests?*

Yes, sorry, we agree that the term is confusing. However, we explicitly wanted to use a summary term, which we could set against the chemical P fertilizer management. We changed the term to “organic P management” and hope this is more straight forward.

11. *Line 279 – where did Bouwman et al. obtain the data? It would be good to know how the data were originally generated originally without having to trace this back through other papers.*

They used data of the International Assessment of Agricultural Knowledge, Science, and Technology for Development as input for the IMAGE model. We included the references in the manuscript (see lines 376­397). We also included information on the original studies of all parameters used in the modelling approach of Ringeval et al. 2017 (please see Table S1).

12. *Line 263 – similarly to my previous comment, I think the authors need to provide more information on the origin of the soil phosphorus data. As written, the reader is required to read multiple other papers to understand the origin of the key data in the current paper. This paper should be able to stand on its own more. It seems unlikely to me that Hedley fractionation data for the spatial scale used in this study has firm empirical grounding. Furthermore, do these soil P estimates take into account the build-up of legacy P over time in cropland soils?*

As described in the manuscript we build on the modelling achievements of Ringeval et al. (2017) and (Bouwman et al., 2006;Bouwman et al., 2013) who used original data from the International Assessment of Agricultural Knowledge, Science and Technology for Developments. Ringeval et al. (2017) simulated the yearly evolution of soil P status during the twentieth century by combining a soil P dynamics model with several global datasets describing the main drivers of P cycle in croplands: soil biogeochemical background, soil inputs/outputs resulting from farm practices (chemical fertilizer, manure, crop uptake, and residues), land use and land cover change, soil temperature and humidity, losses through erosion,

Page 4/9

atmospheric deposition, and soil-order dependent buffering capacity. The soil P model calculates bulk transformations in the first 30 cm with a yearly time step from 1900 to 2005, separating crop and pasture land use at 0.5° resolution globally. Soil P of cropland in 2005 have been used in the current study. Soil biogeochemical background relies on the database of Yang et al., 2013, who built up a global database on P fractions considering major USDA soil taxonomy soil orders, grouped in three soil weathering categories as slightly, intermediately and highly weathered soils. And yes, the modelling approach does take into account the build-up of legacy P over time in cropland soils. We added all this information in the methods section of the manuscript (lines 376-432 and Table S1).

13. *Line 290 – Error estimation – I commend the authors for including this section in their study. Too many manuscripts of this kind do not do so.*

We substantially revised the error estimation and uncertainty assessment section. It is included in the Methods sections of this manuscript (please see lines 433-489). We give detailed information on the uncertainty of soil loss estimates using a Bayesian modelling technique to assess the uncertainty of the different model layers which is then used to fit a Markov Chain Monte Carlo additive model. Regarding the uncertainty of the phosphorus data a similar approach was chosen by Ringeval et al., 2017, however the uncertainty of the model drivers was arbitrarily assumed as 30% and then used for replicated model runs to compute average and standard deviation for each grid cell (please see lines 475-486).

14. *The quality of Figure 3 needs to be improved. Again, I am skeptical of the individual P fractions included here for the global scale.*

We considerably improved the quality of Figure 3. Regarding the individual P fractions, please see our answer to your comment No. 1.

*General comment – there needs to be more discussion in how soil management can impact soil erosion*

*(for better or worse).*

Please see our answer to comment No. 8 above and new lines 347-355.

Answer to Reviewer #2:

*The manuscript entitled ‘P shortage will be aggravated by soil erosion in Europe, Africa, and South America’ pairs two of the co-authors’ datasets on erosion rates and soil P content to create a novel dataset of losses of P through erosion of cropland globally. This is paired with other existing data on agricultural P flows in order to characterize the importance of soil erosion as a loss pathway (which affects both food security and water quality). A spatially explicit dataset of global erosion losses is absolutely an important contribution to the field. As noted by the authors, there are a diversity of global estimates that agree that it is an important loss, but none that really give global coverage while also accounting for smaller spatial scale variations in controlling factors. The article is well written, but I believe a few important pieces of information and contextual comparisons are missing to ensure that all readers can easily and accurately use the results of this study. I realize that one of the biggest challenges with this type of piece can be the word count limit and thus decisions have to be made on what to present (and also at time on the scope of the work). At the same time however, with how high impact some of these global pieces can get, it seems crucial that important findings be properly framed. As such, I support this article being published after these changes have been made (barring any red flags of course from other reviewers with different areas of expertise).*

Thank you for your positive and constructive thoughts and comments and the time you spent on this manuscript.

Page 5/9

*I believe there are 4 areas that warrant a little more nuance and attention (and I apologize in advance if some of this is in the SI material that might not have made it to me. I only saw an SI Figure and not SI text of tables). I have included some line comments in the attached PDF to support these points.*

1) *Explicitly mention the reference year(s) for the databases used and then what is the target year for the numbers given for this analysis.*

Sorry, for not providing this earlier. In Ringeval et al 2017, the model was run over the period 1900-2005 at a time scale of 1 year and was partly based on data from Bouwman et al., 2013. Bouwman provided some input variables ((chemical fertilizer, manure, crop uptake, and residues; please see table S1) for the following years: 1900, 1950, 1960, 1970, 1980, 1990, 1995, 2000 which were linearly extrapolated to compute the input of the model. For the years 2001 - 2005, the data of Bouwman of the year 2000 was used. We included this information in the methods section (line 376 to 397).

2) *In relation to the above, these high loss numbers from erosion (which are quite startling and thus why this paper is likely to be high impact) could be better contextualized with regards to soil P storage, both in terms of legacy P that can be used (or lost) in the future (e.g., Sattari et al. 2012 PNAS, McCrackin et al. 2018 BGC) and also sequestration (e.g., Riskin et al. 2013 philo transactions royal society B). There could also be slightly more nuanced consideration of human and animal excreta recycling, as well as total losses of P to water vs the erosion rates described here.*

Thanks for your comments and we included a paragraph on the importance of different P fractions, the discussion on residual versus non-residual and likely plant available P (please see line 164-184). However, we do feel that discussing the opportunity and limitations of human and animal excreta recycling is going beyond the scope of this manuscript, as others have done so before. We do refer to these studies: “Opportunities for recovering phosphorus and reducing demand have been discussed extensively (e.g., (Cordell et al., 2009;Cordell et al., 2011;Drangert, 1998;Elser and Bennett, 2011))”.

Regarding the startling high values for soil P losses, we would like to point to our chapter “Verification” (lines 189- 238 including Table 3), which shows that we underestimate rather than overestimate P export from soils.

3) *Make datasets, interpretations, and figures easily accessible to all. I was missing links to the full spatially explicit datasets, didn’t see an analysis of uncertainty, and some of the figures and the captions could be clearer (including to color blind people).*

We added a full data availability statement as required by Nature Communications. All data will be available at the European Soil Data Centre (ESDAC). We also added a more detailed description of the uncertainty analysis (see section Error estimation). If the manuscript is accepted for publication, we could provide figures which are comprehensible for colour-blind people in the supporting information (e.g., according to the suggestions of <https://venngage.com/blog/color-blind-friendly-palette/#4>).

Answer to Reviewer #3:

*The objective of the study, to determine spatially distributed P losses from soil erosion for the world, is important and well justified. The approach is based on assumptions and estimates from previous studies that have not previously been combined at this spatial scale, therefore, the novelty is found in the fact these authors are making P loss estimates at a resolution of 250 m. This allows for a more detailed discussion of regions of the world that are experiencing excessive P losses and how this may relate to the sustainability of our P fertilizer reserves. The paper has potential to make a sound contribution to scientific*

Page 6/9

*literature and thinking.*

Thank you for your appreciation.

1. *However, it appears the authors made minor efforts to validate the estimates of their approach. They made a very broad comparison of their estimates with Kronvang et al. (2007). In lines 186 to 196 they concluded that their estimates of 1.2 kg P/ha loss from soil erosion in Europe was on the same order of magnitude with estimates presented by Kronvang et al (2007), which were 0.05 to 3 kg P/ha/yr. However, a closer look at data from Kronvang et al. (2007) shows that the range of erosion estimates was highly skewed by one of the 17 watersheds. The range of P loss from the remaining 16 watersheds was only 0.05 to 1 kg P/ha and the median (for all 17) was 0.41 kg P/ha. Therefore, their estimate of 1.2 kg P/ha is nearly three times greater than the median from the paper they cite.*

We included a full chapter on “Validation P export in comparing soil P loss to riverine exports” (please see line 189 – 238 including Table 3).

And yes, we agree that the Greek catchment might be an outlier in the Kronvang study (thanks for pointing this out) and we thus excluded it from our comparison. If we compare our spatially averaged soil P loss value for Europe (1.2 kg ha^-1^yr^-1^ ) with the range of the remaining 16 catchments by Kronvang et al., (0.05 to 1.5 kg ha^-1^yr^-1^), we are indeed at the higher end of this range. However, in comparing these numbers directly, we compared P loss due to on-site soil erosion risk to off-site P accumulation in the rivers. As on-site erosion potential will always be higher than off-site sediment delivery, we have to assume sediment delivery rates to estimate riverine export from soil loss values. We chose sediment delivery rates between 11-30% as have been recently discussed and applied in large scale (global) studies (Lal et al., 2013; Lugato et al., 2018; Goudie et al., 2013). In doing so, we are actually at the lower range of the estimated values of Kronvang et al. (2007) as well as Beussen et al. (2005) (please see the mentioned verification chapter and Table 3).

2. *Furthermore, there is a wealth of other published data that they could use to validate the estimates from their model. Stackpoole et al. (2019) estimated P balance, including P loss in surface water, for 143 watersheds in the United States. Figure S1 from their paper shows that 75% of all watersheds had less than 1 kg P/ha lost through surface water (which does not account for point sources). Data used by Stackpoole et al. (2019) is available in a published dataset by De Cicco et al. (2017). Baker et al. (2014) determined P losses from three major watersheds draining into lake Erie in North America and, after subtracting out P from point-source discharges, the P export in river due to non-point source was between 1 and 1.3 kg P/ha/yr. The authors estimate that P loss due to erosion in North America is 4.6 kg P/ha/yr, which is over 4 times the estimates from above cited sources of measured data.*

*It is important to note that the authors state their model does not calculate the net catchment output, but rather on-site displacement of soils (lines 136-138), however, they should be able to use published information about catchment delivery ratios to determine if their estimates are valid relative to data on published catchment-scale P losses. I recommend the authors present additional data related to validation of estimates produced by their modeling approach compared to available measured data.*

To validate our on-site P soil loss approach with off-site P river export data requires two prerequisites: (i) an estimation of the agricultural erosion and runoff contribution to total P loads in the rivers and (ii) an assumption on sediment delivery rates to rivers to estimate P loads to rivers from on-site P soil loss. There is a lack of models describing integrated sediment-delivery to rivers on a continental or global scale , but it has been discussed that sediment delivery ratios generally decrease as drainage area increases, ranging from roughly 30-100% in small catchment (≤ 0.1 km^2^) to 2 – 20% at large spatial scales (e.g., ≥ 1000 km^2^) (Goudie, 2013). For our comparison, we used a range of average sediment delivery rates between 11 – 30% as used in recent large-scale studies from continental to global scale (Lal et al., 2003; Lugato et al., 2018). A comparison of calculated potential P export to rivers from our on-site soil P losses is well within the ranged of published riverine P exports (Table 3). The river loads calculated by Stackpoole et al. (2019)

Page 7/9

match very well with our assessments (0.3 -1 versus 0.5 to 1.4 kg ha^-1^yr^-1^, respectively) while P loads to Lake Erie (Baker et al., 2014) are considerably higher than the continental average (Table 3). However, no partitioning between agricultural, urban or industrial fluxes was possible from the latter study. (Sorry, we could not find the value 1 – 1.3 kg ha^-1^yr^-1^ in the Baker study. Was this the value related to agricultural runoff? Than this would actually match our values perfectly).

*Editorial comments*

*Line 65-66: Please note that Asia also has a positive balance in Table 1.*

yes, sorry, we corrected to “...with Asia having a slightly positive but near zero P balance.”

*Table 1: correct spelling of “Amerika” to “America”*

done

References

Baker, D. B., Confesor, R., Ewing, D. E., Johnson, L. T., Kramer, J. W., and Merryfield, B. J.: Phosphorus loading to Lake Erie from the Maumee, Sandusky and Cuyahoga rivers: The importance of bioavailability, Journal of Great Lakes Research, 40, 502-517, 10.1016/j.jglr.2014.05.001, 2014.

Beusen, A. H. W., Dekkers, A. L. M., Bouwman, A. F., Ludwig, W., and Harrison, J.: Estimation of global

river transport of sediments and associated particulate C, N, and P, Global Biogeochemical Cycles, 19, 19, 10.1029/2005gb002453, 2005.

Borbor-Cordova, M. J., Boyer, E. W., McDowell, W. H., and Hall, C. A.: Nitrogen and phosphorus budgets for a tropical watershed impacted by agricultural land use: Guayas, Ecuador, Biogeochemistry, 79, 135-161, 10.1007/s10533-006-9009-7, 2006.

Borrelli, P., Robinson, D. A., Fleischer, L. R., Lugato, E., Ballabio, C., Alewell, C., Meusburger, K., Modugno, S., Schütt, B., Ferro, V., Bagarello, V., Oost, K. V., Montanarella, L., and Panagos, P.: An assessment of the global impact of 21st century land use change on soil erosion, Nature Communications, 8, 2013, 10.1038/s41467-017-02142-7, 2017.

Bouwman, A., Kram, T., and Klein Goldewijk, K.: Integrated modelling of global environmental change, An overview of IMAGE, 2, 225-228, 2006.

Bouwman, L., Goldewijk, K. K., Van Der Hoek, K. W., Beusen, A. H., Van Vuuren, D. P., Willems, J., Rufino, M. C., and Stehfest, E.: Exploring global changes in nitrogen and phosphorus cycles in agriculture induced by livestock production over the 1900–2050 period, Proceedings of the National Academy of Sciences, 110, 20882-20887, 2013.

Cordell, D., Drangert, J. O., and White, S.: The story of phosphorus: Global food security and food for thought, Global Environmental Change-Human and Policy Dimensions, 19, 292-305, 10.1016/j.gloenvcha.2008.10.009, 2009.

Cordell, D., Rosemarin, A., Schroder, J. J., and Smit, A. L.: Towards global phosphorus security: A systems framework for phosphorus recovery and reuse options, Chemosphere, 84, 747-758, 10.1016/j.chemosphere.2011.02.032, 2011.

Drangert, J. O.: Fighting the urine blindness to provide more sanitation options, Water Sa, 24, 157-164, 1998.

Elser, J., and Bennett, E.: A broken biogeochemical cycle, Nature, 478, 29-31, 2011.

Goudie, A.: Encyclopedia of Geomorphology, Taylor & Francis, 2013.

Helfenstein, J., Pistocchi, C., Oberson, A., Tamburini, F., Goll, D. S., and Frossard, E.: Estimates of mean residence times of phosphorus in commonly considered inorganic soil phosphorus pools, Biogeosciences, 17, 441-454, 10.5194/bg-17-441-2020, 2020.

Koci, J., Sidle, R. C., Kinsey-Henderson, A. E., Bartley, R., Wilkinson, S. N., Hawdon, A. A., Jarihani, B., Roth, C. H., and Hogarth, L.: Effect of reduced grazing pressure on sediment and nutrient yields in savanna rangeland streams draining to the Great Barrier Reef, J. Hydrol., 582, 14, 10.1016/j.jhydrol.2019.124520, 2020.

Page 8/9

Kronvang, B., Vagstad, N., Behrendt, H., Bogestrand, J., and Larsen, S. E.: Phosphorus losses at the catchment scale within Europe: an overview, Soil Use Manage., 23, 104-116, 10.1111/j.1475-2743.2007.00113.x, 2007.

Lal, R.: Soil erosion and the global carbon budget, Environment International, 29, 437-450,

10.1016/s0160-4120(02)00192-7, 2003.

Liu, Y., Villalba, G., Ayres, R. U., and Schroder, H.: Global phosphorus flows and environmental impacts from a consumption perspective, Journal of Industrial Ecology, 12, 229-247, 10.1111/j.1530-9290.2008.00025.x, 2008.

Lugato, E., Smith, P., Borrelli, P., Panagos, P., Ballabio, C., Orgiazzi, A., Fernandez-Ugalde, O., Montanarella, L., and Jones, A.: Soil erosion is unlikely to drive a future carbon sink in Europe, Science Advances, 4, 10.1126/sciadv.aau3523, 2018.

Ringeval, B., Augusto, L., Monod, H., van Apeldoorn, D., Bouwman, L., Yang, X., Achat, D. L., Chini, L. P., Van Oost, K., Guenet, B., Wang, R., Decharme, B., Nesme, T., and Pellerin, S.: Phosphorus in agricultural soils: drivers of its distribution at the global scale, Global Change Biology, n/a-n/a, 10.1111/gcb.13618, 2017.

Riskin, S. H., Porder, S., Neill, C., Figueira, A., Tubbesing, C., and Mahowald, N.: The fate of phosphorus fertilizer in Amazon soya bean fields, Philosophical Transactions of the Royal Society B-Biological Sciences, 368, 10.1098/rstb.2012.0154, 2013.

Sattari, S. Z., Bouwman, A. F., Giller, K. E., and van Ittersum, M. K.: Residual soil phosphorus as the missing piece in the global phosphorus crisis puzzle, Proceedings of the National Academy of Sciences of the United States of America, 109, 6348-6353, 10.1073/pnas.1113675109, 2012.

Stackpoole, S. M., Stets, E. G., and Sprague, L. A.: Variable impacts of contemporary versus legacy

agricultural phosphorus on US river water quality, Proceedings of the National Academy of Sciences, 116, 20562-20567, 10.1073/pnas.1903226116, 2019.

Stoorvogel, J. J., Smaling, E. M. A., and Janssen, B. H.: CALCULATING SOIL NUTRIENT BALANCES IN AFRICA AT DIFFERENT SCALES .1. SUPRA-NATIONAL SCALE, Fertilizer Research, 35, 227­235, 10.1007/bf00750641, 1993.

Tiessen, H., Stewart, J. W. B., and Cole, C. V.: PATHWAYS OF PHOSPHORUS TRANSFORMATIONS IN SOILS OF DIFFERING PEDOGENESIS, Soil Science Society of America Journal, 48, 853­858, 10.2136/sssaj1984.03615995004800040031x, 1984.

Wang, X., Hao, F. H., Cheng, H. G., Yang, S. T., Zhang, X., and Bu, Q. S.: Estimating non-point source pollutant loads for the large-scale basin of the Yangtze River in China, Environ. Earth Sci., 63,

1079-1092, 10.1007/s12665-010-0783-0, 2011.

Yang, X., Post, W. M., Thornton, P. E., and Jain, A.: The distribution of soil phosphorus for global biogeochem ical modeling, Biogeosciences, 10, 2525-2537, 10.5194/bg-10-2525-2013, 2013.

Zehetner, F., Wuenscher, R., Peticzka, R., and Unterfrauner, H.: Correlation of extractable soil phosphorus (P) with plant P uptake: 14 extraction methods applied to 50 agricultural soils from Central Europe, Plant Soil and Environment, 64, 192-201, 10.17221/70/2018-pse, 2018

**Reviewer comments, second version:**

Reviewer #1 (Remarks to the Author):

The authors have obviously invested substantial time in revising this manuscript. I think these efforts have paid off and that the new version is much improved. However, I have some additional comments and a lingering concern. These are described below:

Table 1 - Specify what "organic P management" means in the legend. I think there is some risk of misunderstanding with the use of this term, given the meaning of "organic management" in the broader food systems literature. But as long as the authors clearly define the term in the text and in the table, then I think it is okay.

Table 1 – While I agree with the authors that it is important to recognize that not all P lost via erosion would have been plant-available, I still have reservations about including different soil P pools for this scale of analysis in the way presented. I appreciate that the authors took time to explain P fractionation schemes in their response. I am very familiar with these schemes (in the lab and in the literature), which is precisely why I have reservations. I think the discussion of this topic in the manuscript remains problematic. For example, I challenge the suggestion in Line 157 that the Riskin et al. 2013 paper shows "convincing evidence" that apatite or occluded P fractions are likely to be at least partly plant available. The fact that P is largely accumulating in the NaOH inorganic P pool in P-sorbing soils receiving high P fertilizer inputs in Brazil (the primary conclusion of Riskin et al 2013) does not provide evidence that 1 M HCl and residual pools (i.e., apatite or occluded P) are in reality partly plant available. They may be to some degree, but the Riskin et al. 2013 study did not test that. The other paper cited (Sattari et al 2012) is a large scale modeling paper and does not itself provide direct evidence of individual soil P fractions becoming plant available. Ultimately, as my comments here illustrate, I still think the degree to which P fractions are discussed and presented in Table 1, Figure 3, and the text distracts from the main goals of this paper. I think a cleaner (i.e., less problematic) approach would be to only report across continents and regions:
(a) the estimates of total P loss, and
(b) the estimated proportion of this P that would have otherwise been available to crops (citing the Hedley methodology once to define how this proportion was calculated)
Without including data for individual P fractions in Table 1, Figure 3, or the related discussion.

Furthermore, I am having trouble determining whether the P soil loss estimates and River load estimates for this study in Table 3 are based on total P or the potentially bioavailable P metric used in Figure 2 (not including apatite or occluded P). Can the authors please clarify? If they used the latter, are these comparisons to regional studies in Table 3 “apples to apples”? In other words, are the numbers from other regional studies in Table 3 based on total P or the P fractions included in Figure 2? Does this affect the verification of the model results?

Reviewer #2 (Remarks to the Author):

The authors of ‘P shortage will be aggravated by soil erosion in Europe, Africa, and South America’ have improved the manuscript by incorporating comments by the various reviewers but I remain unsatisfied with how they have dealt with a small subset of the issues raised. Of course, I believe the authors can address these issues, as I thought in the previous round, and thus that the paper will be relevant to publish as it is a very nice piece of work!

Nutrient budgets and accounting for recycling:

In the end of introduction, I felt that there was still an issue with how the authors juxtapose erosion with other agricultural P flows. Closing the cycle with recycling can address some of the scarcity and pollution if well management to match plant demand but if erosion is high, then losses are high regardless of if the P is from manure or chemical fertilizer (I realize the forms are different and have different implications but I am speaking in broad terms here).

As such I agree with the authors that accounting for erosion is key, but the hypothesis seems to be missing a part of its importance here.

Bringing up nutrient reuse (or legacy P) in the discussion I would argue is not beyond the scope of this work. I agree that dealing with it in a more detailed way in the methods would be a beyond the scope, but acknowledging the ways in which the base data used, and the equations those data are put in, in this work, not about only erosion, but the other P flows that loss is compared to, is essential. The over and under application of manure and other organic waste products due to a diversity of issues (e.g., distance) affects where and how losses from soil occur as well as where fertilizer is needed. As such the resolution of each data set matters immensely in explaining results. On page 12 and 13 there is indeed good material discussing P balances, but there is a big difference in doing that at a country level, a state level, a farm level, or in a spatially explicit gridded dataset.

A more specific example would be line 272 page 12. Yes increasing recycling can help many parts of Africa but there could be 2 issues: 1) low P in soils limiting crop production so even if farmers are recycling everything locally it’s not enough to boost yields, and 2) bringing in recycled materials from other parts of the word is difficult (possible of course but requires processing). Depending on the base data used in this study this could then interact in a number of ways in how to interpret results. For instance, if the soil P concentrations in Africa increase with increased recycling and synthetic fertilizer use then in fact erosion control because more important from an aquatic perspective, even if maybe locally there is less worry about the P availability side.


Visual presentation and accessibility:

I am unsatisfied with the answer that if accepted the figures will be changed to be more accessible. This should be done as part of the review process. Green to red color schemes are not just bad for color blind people, they also lead to a natural human reaction to break the dataset at yellow values when in fact the datasets presented here are continuous (and above 0 so it should be 1 continuous color). Humans are also quite bad at estimating area which makes piechart often a subpar choice for visualization. I do realize the numbers are also put on the charts which is great, but sharing and coloring of words in the boxes can make things hard to read. In most cases the boxes are not needed, just the text and arrows (and numbers), and maybe they could be organized so they are easier to compare between world regions?

I remained a bit confused about the data availability statement, but maybe the confusion is on my end. The authors put forward that the data is freely accessible which is wonderful of course, but my understanding is the authors transformed these base datasets by combining the 2 data papers mentioned in the manuscript and I believe making the actual data used in the paper is important for the results to be useful. To me that means providing the values (and code when applicable) to make the figures to increase the usability of this study to authors who might want to use these insights at a regional level for instance. Table S1 was quite helpful, but it would be great to go a step further.


Small points:

Page 3 line 82 missing an and

Page 3 line 86 strange phrasing to begin the sentences. I felt confused if Africa had the biggest losses as a percentage of inputs or it total or something else.

Page 3 line 97 Hard to follow. So what year is this for? Is it an average over the full time series? is it for the future? is it today?

Page 17 lune 426 realizations


I look forward to this article being out :)


Reviewer #3 (Remarks to the Author):

The authors did a very good job of clearly describing how their model estimates compare to estimates from other published studies. They clearly state that the comparison is heavily dependent on the delivery ratios, but they are transparent in their comparison, which allows readers to make their own independent assessment. I greatly appreciate the authors attention to responding to this comment.

No further revisions of the paper are necessary.

As an aside, I found the value of 1 to 1.3 kg ha-1 yr-1 of P loss in tables 7 and 2 in Baker et al., 2014.

From table 7, annual P loss in 2012 was 2361, 629, and 284 Mg in the Maumee, Sandusky, and Cuyahoga watersheds. From Table 2 the drainage area is 16388, 3289, and 1830 km2 for the Maumee, Sandusky, and Cuyahoga. Which would result in 1.44, 1.91, and 1.55 kg P ha-1 loss. At the bottom of the first page of the paper they state that non-point sources account for 67% of the P load, therefore, non-point loads from these watersheds would be roughly 1 to 1.3 kg P ha-1.

There may be a better value to cite elsewhere in the paper.

**Author rebuttal, second version:**

**REVIEWER COMMENTS**

**Reviewer #1 (Remarks to the Author):**

*The authors have obviously invested substantial time in revising this manuscript. I think these efforts have paid off and that the new version is much improved. However, I have some additional comments and a lingering concern. These are described below.*

Thank you for your appreciation and also for your time and effort to improve this manuscript. We carefully considered all comments. Please find our answers/ revisions below and note that all line references refer to the track changes version of our manuscript. Please also note that only changes of this second revision are visualised in this document.

*Table 1 - Specify what "organic P management" means in the legend. I think there is some risk of misunderstanding with the use of this term, given the meaning of "organic management" in the broader food systems literature. But as long as the authors clearly define the term in the text and in the table, then I think it is okay.*

We apologize for not providing the legend in Table 1. We did so in Figure 3 but forgot in Table 1 in the first revision but added it now in our second revision.

*Table 1 – While I agree with the authors that it is important to recognize that not all P lost via erosion would have been plant-available, I still have reservations about including different soil P pools for this scale of analysis in the way presented. I appreciate that the authors took time to explain P fractionation schemes in their response. I am very familiar with these schemes (in the lab and in the literature), which is*

Page 1/5

University of Basel Environmental Geosciences Bernoullistrasse 30 4056 Basel, Switzerland [umweltgeo.unibas.ch](http://umweltgeo.unibas.ch)

Christine Alewell

T +41 61 207 04 77

[Christine.alewell@unibas.ch](mailto:Christine.alewell@unibas.ch)

*precisely why I have reservations. I think the discussion of this topic in the manuscript remains problematic. For example, I challenge the suggestion in Line 157 that the Riskin et al. 2013 paper shows "convincing evidence" that apatite or occluded P fractions are likely to be at least partly plant available. The fact that P is largely accumulating in the NaOH inorganic P pool in P-sorbing soils receiving high P fertilizer inputs in Brazil (the primary conclusion of Riskin et al 2013) does not provide evidence that 1 M HCl and residual pools (i.e.,*

*apatite or occluded P) are in reality partly plant available. They may be to some degree, but the Riskin et al. 2013 study did not test that. The other paper cited (Sattari et al 2012) is a large scale modeling paper and does not itself provide direct evidence of individual soil P fractions becoming plant available.*

We agree with the fact that the initially used references (Riskin et al.,2013 ; Sattari et al., 2012) were not appropriate to explain that apatite and occluded P previously considered as not plant available may in fact be used by plants at medium time scale. We removed these references and rely further on Helfenstein et al (2018, 2020) as references, which aim to quantify the residence time of each Hedley fractions as well as to provide information about the underlying biological/biogeochemical processes.

*Ultimately, as my comments here illustrate, I still think the degree to which P fractions are discussed and presented in Table 1, Figure 3, and the text distracts from the main goals of this paper. I think a cleaner (i.e., less problematic) approach would be to only report across continents and regions:*

(a) *the estimates of total P loss, and*
(b) *the estimated proportion of this P that would have otherwise been available to crops (citing*

*the Hedley methodology once to define how this proportion was calculated)*

*Without including data for individual P fractions in Table 1, Figure 3, or the related discussion.*

Thanks for this suggestion, which is indeed a very good solution. We followed this concept and refer to plant available and non-plant available in Figure 3. We clearly defined in the Methods (lines 433-450) what we called plant available / non-plant available in our manuscript (by putting together some Hedley fractions), as the definition of plant available P is prone to uncertainty.We did insert the subfractions of organic and inorganic plant available P but deleted apatite and occluded P fractions. We also changed the related text which read as follows in the new version (lines 154-168):

“Only a small fraction of the total soil P is plant available, because a large fraction is either bound in, adsorbed to or made unavailable by occlusion in minerals (apatite and occluded P)^41^. Our modelling approach follows the approach of Yang, et al. ^41^ that builds on existing knowledge of soil P processes and data bases to provide spatially explicit estimates of different forms of naturally occurring soil P on the global scale. Yang, et al. ^41^ uses data acquired with the Hedley fractionation method^42^ which splits soil P into different fractions that are extracted sequentially with successively stronger reagents and which are merged into various inorganic and organic pools. There is great uncertainty to associate these fractions with functional plant uptake^43,44^ but some recent works aims at quantifying residence times of the different fractions^17,45^. We present the total P loss as well as the plant-available pool (Figures 3 and 4, Table 1) as the most labile P which can participate to plant nutrition in a time scale up to months^17^ (i.e. the so-called labile inorganic P, inorganic P bound to secondary minerals, labile and stable organic P in ^33,41).^ We contrast these more labile, short lived fractions with fractions considered very stable which would not be plant available at short time scales (i.e., inorganic P associated with minerals such as apatite or occluded P^17^, corresponding to 52 % of total soil P in our estimates at the global scale).”

*Furthermore, I am having trouble determining whether the P soil loss estimates and River load estimates for this study in Table 3 are based on total P or the potentially bioavailable P metric used in Figure 2 (not including apatite or occluded P). Can the authors please clarify? If they used the latter, are these comparisons to regional studies in Table 3 “apples to apples”? In other words, are the numbers from other regional studies in Table 3 based on total P or the P fractions included in Figure 2? Does this affect the verification of the model results?*

Page 2/5

We indeed considered total P (please compare Table 1 and Table 3) in applying the sediment delivery rates and comparing to riverine loads. We assumed that quantifying the P loads in rivers would also target on total suspended P.

**Reviewer #2 (Remarks to the Author):**

*The authors of ‘P shortage will be aggravated by soil erosion in Europe, Africa, and South America’ have improved the manuscript by incorporating comments by the various reviewers but I remain unsatisfied with how they have dealt with a small subset of the issues raised. Of course, I believe the authors can address these issues, as I thought in the previous round, and thus that the paper will be relevant to publish as it is a very nice piece of work!*

Thanks a lot for your comments, time and effort to improve our manuscript, and, indeed, your

appreciation. Please see below for our detailed response and comments and note that all line references refer to the track changes version of our manuscript. Please also note that only changes of this second revision are visualised in this document.

*Nutrient budgets and accounting for recycling:*

*In the end of introduction, I felt that there was still an issue with how the authors juxtapose erosion with other agricultural P flows. Closing the cycle with recycling can address some of the scarcity and pollution if well management to match plant demand but if erosion is high, then losses are high regardless of if the P is from manure or chemical fertilizer (I realize the forms are different and have different implications but I am speaking in broad terms here).*

*As such I agree with the authors that accounting for erosion is key, but the hypothesis seems to be missing a part of its importance here.*

*Bringing up nutrient reuse (or legacy P) in the discussion I would argue is not beyond the scope of this work. I agree that dealing with it in a more detailed way in the methods would be a beyond the scope, but acknowledging the ways in which the base data used, and the equations those data are put in, in this work, not about only erosion, but the other P flows that loss is compared to, is essential. The over and under application of manure and other organic waste products due to a diversity of issues (e.g., distance) affects where and how losses from soil occur as well as where fertilizer is needed. As such the resolution of each data set matters immensely in explaining results. On page 12 and 13 there is indeed good material discussing P balances, but there is a big difference in doing that at a country level, a state level, a farm level, or in a spatially explicit gridded dataset.*

We totally agree with you, that P loss due to erosion is only one part of the problem and that it is crucial to consider if global, national, regional or local budgets and cycles are discussed. We targeted on a global/ continental scale. However, to follow your idea, we inserted discussion on one African country, Malawi as (i) Africa is for sure the continent with the most pressing problems regarding nutrient deficiencies and P scarcity now and in the future and (ii) Malawi seems to be a country having adopted promising strategies to tackle the problem. Even if Malawi might not be there yet to fully solve the problems, it has certainly put considerable effort with quite some success in solution approaches. Please see our text additions line 293 to 324.

*A more specific example would be line 272 page 12. Yes increasing recycling can help many parts of Africa but there could be 2 issues: 1) low P in soils limiting crop production so even if farmers are recycling everything locally it’s not enough to boost yields, and 2) bringing in recycled materials from other parts of the word is difficult (possible of course but requires processing). Depending on the base data used in this study this could then interact in a number of ways in how to interpret results. For instance, if the soil P concentrations in Africa increase with increased recycling and synthetic fertilizer use then in fact erosion*

Page 3/5

*control because more important from an aquatic perspective, even if maybe locally there is less worry about the P availability side.*

Yes, we fully agree that if soil P pools are already very low, African farmers will run into P deficiencies even without erosion. And, as we wrote in the manuscript it will be very difficult to increase residue and manure P input in African systems. We discussed options including growing legumes as cover crops and generally reformulated to indicate that erosion control/ mitigation is only part of the solution and that even a total stop of erosion might not help to avoid P deficiency if other measures are not adopted (please see line 293-324).

*I am unsatisfied with the answer that if accepted the figures will be changed to be more accessible. This should be done as part of the review process. Green to red color schemes are not just bad for color blind people, they also lead to a natural human reaction to break the dataset at yellow values when in fact the datasets presented here are continuous (and above 0 so it should be 1 continuous color). Humans are also quite bad at estimating area which makes piechart often a subpar choice for visualization. I do realize the numbers are also put on the charts which is great, but sharing and coloring of words in the boxes can make things hard to read. In most cases the boxes are not needed, just the text and arrows (and numbers), and maybe they could be organized so they are easier to compare between world regions?* We changed all Figures and followed the Nature Communications recommendations regarding colour scales in Fig. 1 to 4 and in addition revised Figure 3 and 4 substantially.

*I remained a bit confused about the data availability statement, but maybe the confusion is on my end. The authors put forward that the data is freely accessible which is wonderful of course, but my understanding is the authors transformed these base datasets by combining the 2 data papers mentioned in the manuscript and I believe making the actual data used in the paper is important for the results to be useful. To me that means providing the values (and code when applicable) to make the figures to increase the usability of this study to authors who might want to use these insights at a regional level for instance. Table S1 was quite helpful, but it would be great to go a step further.*

We included a full data availability checklist in our manuscript:

“The data presented in this study (Fig. 1 and 2) as well as all input layers to our modelling approach are directly available at the European Soil Data Centre (ESDAC) of the European Commission – Joint Research Centre: [https://esdac.jrc.ec.europa.eu/themes/global-phosphorus](https://Access). Access to the data is free for all public users.” (lines 483 as well as 564 ff)

The web page is already set up and online. We will make the data available as soon as the manuscript is accepted. Regarding the modelling code, we presented all necessary equations in the methods sections.

*Small points:*

*Page 3 line 82 missing an and*

*Sorry, not sure what is meant, I copy below the original manuscript lines:*

*Page 3 line 86 strange phrasing to begin the sentences. I felt confused if Africa had the biggest losses as a percentage of inputs or it total or something else.*

Sorry, yes this was unclear. We changed to. “Most negative P balances are indicated to for Africa...” (line 91).

Page 4/5

*Page 3 line 97 Hard to follow. So what year is this for? Is it an average over the full time series? is it for the future? is it today?*

This is a hypothetical scenario, if all countries would run out of phosphorous, meaning there is no chemical phosphorous fertilizer available any more. We changed the beginning of this paragraph to “With the hypothetical assumption of no replenishment due to chemical fertilizer (e.g., due to economic or technical constraints)...” (line 99)

*Page 17 lune 426 realizations*
changed, thank you

*I look forward to this article being out :)*
Thank you! We too, ☺

**Reviewer #3 (Remarks to the Author):**

*The authors did a very good job of clearly describing how their model estimates compare to estimates from other published studies. They clearly state that the comparison is heavily dependent on the delivery ratios, but they are transparent in their comparison, which allows readers to make their own independent assessment. I greatly appreciate the authors attention to responding to this comment..*

Thank you, appreciated

*No further revisions of the paper are necessary*

*As an aside, I found the value of 1 to 1.3 kg ha-1 yr-1 of P loss in tables 7 and 2 in Baker et al., 2014. From table 7, annual P loss in 2012 was 2361, 629, and 284 Mg in the Maumee, Sandusky, and Cuyahoga watersheds. From Table 2 the drainage area is 16388, 3289, and 1830 km2 for the Maumee, Sandusky, and Cuyahoga. Which would result in 1.44, 1.91, and 1.55 kg P ha-1 loss. At the bottom of the first page of the paper they state that non-point sources account for 67% of the P load, therefore, non-point loads from these watersheds would be roughly 1 to 1.3 kg P ha-1. There may be a better value to cite elsewhere in the paper.*

You are right, thanks for pointing this out. The average of the three catchments is 1.6 kg ha^-1^yr^-1^ (which compares to our values actually even better).

Baker et al.. 2014

loads

area (km^2^)	tons yr^-1^

catchment	(Table 2)	(Table 7)	kg ha^-1^yr^-1^

Maumee	16,388	2,361	1.4

Sandusky	3,289	629	1.9

Cuyahoga	1,830	284	1.6

average	1.6

**Reviewer comments, third version:**

Reviewer #1 (Remarks to the Author):

The authors have done a great job addressing my previous comments. I have no further concerns.


Reviewer #2 (Remarks to the Author):

The authors have done a good job addressing this round of comments.

I still feel that the point that future fertiliser application will interact with erosion is not highlighted (aka actually making erosion control more important because there will be more P in the soil that can be lost) but I think enough is now said for the reader to contextualise the authors' findings.

Having worked in Malawi a little myself, I can say that the fertiliser subsidy program results in very patchy yield increases (you can literally see which farmers got the fertiliser bags that year) and erosion control will continue to be a very difficult issue to address as maize is grown on marginal sloping lands.

**Author rebuttal, third version:**

**Reviewer #1 (Remarks to the Author):**

*The authors have done a great job addressing my previous comments. I have no further concerns.* Thanks you for your appreciation.

**Reviewer #2 (Remarks to the Author):**

*The authors have done a good job addressing this round of comments.*
Thanks you for your appreciation.

*I still feel that the point that future fertiliser application will interact with erosion is not highlighted (aka actually making erosion control more important because there will be more P in the soil that can be lost) but I think enough is now said for the reader to contextualise the authors' findings.*

We do think we addressed this point several times in pointing to (i) the importance of soil erosion control with increasing deficiency in P fertiliser for physical, economical or technical reasons and (ii) impacts of eutrophication on biodiversity in adjacent ecosystems and waters with high P export due to soil erosion. The thought that there will be more P in the soil that will be lost in the future would imply that increasing amounts of P fertiliser might be used in the future, which might go beyond the scope of this manuscript (e.g., addressing possible future management scenarios).

*Having worked in Malawi a little myself, I can say that the fertiliser subsidy program results in very patchy yield increases (you can literally see which farmers got the fertiliser bags that year) and erosion control will continue to be a very difficult issue to address as maize is grown on marginal sloping lands.*

Yes, we fully agree with this statement and have thus described the complex picture of possible land management in Malawi in lines 266-292
